# Surface functionalization and size modulate the formation of reactive oxygen species and genotoxic effects of cellulose nanofibrils

**DOI:** 10.1186/s12989-022-00460-3

**Published:** 2022-03-16

**Authors:** Kukka Aimonen, Monireh Imani, Mira Hartikainen, Satu Suhonen, Esa Vanhala, Carlos Moreno, Orlando J. Rojas, Hannu Norppa, Julia Catalán

**Affiliations:** 1 Finnish Institute of Occupational Health, Työterveyslaitos, Box 40, 00032 Helsinki, Finland; 2 Department of Bioproducts and Biosystems, Aalto University, Espoo, Finland; 3 Department of Anatomy, Embryology and Genetics, University of Zaragoza, Zaragoza, Spain; 4 Bioproducts Institute, Departments of Chemical and Biological Engineering, Chemistry and Wood Science, The University of British Columbia, Vancouver, BC Canada

**Keywords:** Cellulose nanofibrils, Nanofibrillated celluloses, High aspect ratio, Functionalization, Surface chemistry, Genotoxicity, Reactive oxygen species

## Abstract

**Background:**

Cellulose nanofibrils (CNFs) have emerged as a sustainable and environmentally friendly option for a broad range of applications. The fibrous nature and high biopersistence of CNFs call for a thorough toxicity assessment, but it is presently unclear which physico-chemical properties could play a role in determining the potential toxic response to CNF. Here, we assessed whether surface composition and size could modulate the genotoxicity of CNFs in human bronchial epithelial BEAS-2B cells. We examined three size fractions (fine, medium and coarse) of four CNFs with different surface chemistry: unmodified (U-CNF) and functionalized with 2,2,6,6-tetramethyl-piperidin-1-oxyl (TEMPO) (T-CNF), carboxymethyl (C-CNF) and epoxypropyltrimethylammonium chloride (EPTMAC) (E-CNF). In addition, the source fibre was also evaluated as a non-nanosized material.

**Results:**

The presence of the surface charged groups in the functionalized CNF samples resulted in higher amounts of individual nanofibrils and less aggregation compared with the U-CNF. T-CNF was the most homogenous, in agreement with its high surface group density. However, the colloidal stability of all the CNF samples dropped when dispersed in cell culture medium, especially in the case of T-CNF. CNF was internalized by a minority of BEAS-2B cells. No remarkable cytotoxic effects were induced by any of the cellulosic materials. All cellulosic materials, except the medium fraction of U-CNF, induced a dose-dependent intracellular formation of reactive oxygen species (ROS). The fine fraction of E-CNF, which induced DNA damage (measured by the comet assay) and chromosome damage (measured by the micronucleus assay), and the coarse fraction of C-CNF, which produced chromosome damage, also showed the most effective induction of ROS in their respective size fractions.

**Conclusions:**

Surface chemistry and size modulate the in vitro intracellular ROS formation and the induction of genotoxic effects by fibrillated celluloses. One cationic (fine E-CNF) and one anionic (coarse C-CNF) CNF showed primary genotoxic effects, possibly partly through ROS generation. However, the conclusions cannot be generalized to all types of CNFs, as the synthesis process and the dispersion method used for testing affect their physico-chemical properties and, hence, their toxic effects.

**Supplementary Information:**

The online version contains supplementary material available at 10.1186/s12989-022-00460-3.

## Background

Cellulose nanomaterials have emerged as sustainable and environmentally friendly options for a broad range of applications, such as rheology modification, coatings and adhesives, composites, foams, hydrogels and aerogels, energy storage systems, packaging, electronic devices and biomedical devices, among others [1–5]. Cellulosic fibres produced from wood and other sources can be fractionated into fibrils of various lateral dimensions (ranging from less than 100 µm to around 2–4 nm), mostly composed of cellulose [6]. The term ‘fibrillated cellulose’ is generally used to refer to cellulose fibres deconstructed into smaller fibrils [5], including cellulose microfibres, and cellulose nanofibrils (CNF; also known as nanofibrillated cellulose) [6–8], as well as highly heterogenous combinations of such morphologies [9], and those with more complex chemical composition, including lignocellulose nanofibres [10]. Fibrillated celluloses are typically produced by mechanical fibrillation by using, e.g., homogenizers, fluidizers, microgrinders and aqueous counter collision systems [11, 12]. Prior to fibrillation, various chemical and enzymatic pre- treatments can be applied to ease the deconstruction into homogeneous fibril dispersions [6, 13]. Fibrillated celluloses are composed of amorphous and crystalline domains [14] and are characterized by a diameter of 3–100 nm and lengths in the micrometer-scale (> 1 µm) [15].

With the expected increasing presence of cellulose nanomaterials in the market, it becomes a priority to assess the safety profile of these materials [17, 18]. The biopersistence [19–22] and high aspect ratio of fibrillated celluloses raise the question of possible effects on human health [17], especially if inhaled as air-borne materials. Inhalation is the main exposure route in occupational settings [18], for instance, during the spraying processes applied to obtain high performance nanocomposites for barrier applications and smooth films for electronics [23].

The size of a nanomaterial is of major importance in determining its hazardous effects [24]. The aspect ratio is considered relevant for the inhalation toxicity of fibrous materials. According to the fibre paradigm hypothesis, fibre-like structures combined with biopersistence may cause fibrosis and malignancy upon inhalation [24].

Long fibres (> 5 µm) may be more difficult to clear than shorter fibres and may persist inside the lungs for a longer time, which can lead to chronic inflammation [25]. Besides, the diameter of nanofibres may also influence their toxicological profile, as reported for some multi-walled carbon nanotubes (MWCNTs) [26, 27].

Surface chemistry has also been considered a key feature modulating toxicological response to nanomaterials [24]. Surface chemistry is critically important in determining how cellulose nanomaterials interact with their environment, dictating their colloidal stability and rheological and interfacial properties, thus affecting all ensuing uses of these materials [4, 8]. Therefore, various chemistries have been developed aiming at modifying the surface of cellulose nanomaterials, e.g., to confer them with specific properties and extend their use to a broader range of applications [28]. Chemical pre-treatments introduce, for example, carboxyl, carboxymethyl, aldehyde or phosphoryl groups on the surface of CNF [29]. Pretreatments also affect the dimensions, colloidal stability, specific surface area and degree of branching of nanofibril structures [30].

The potential adverse health effects of cellulose nanomaterials have increasingly been studied during the last few years [4, 16, 31–33]. In the case of CNF, toxicological studies have shown contradictory findings. This may partly be due to the broad range of CNF properties mainly arising from the fibre source, the extraction/production method, and the surface chemistry [8]. With the exception of a few studies assessing the toxicological potential of cellulose nanofibrils that only differ in their surface chemistry [34–36], most of the reported attempts to compare different CNF types have been challenged by the many variables affecting the results [20–22, 37].

To date, only a few in vivo studies have assessed the pulmonary effects of CNFs, after (oro)pharyngeal aspiration or intratracheal instillation in mice [20–22, 38, 39]. The studies described an acute inflammatory response which subsided in 28 days [21, 22, 38] and differentiation of T-cells toward a Th1-phenotype at 14 days post-exposure [39]. Furthermore, genotoxic effects (DNA damage) were observed in the lungs or bronchoalveolar lavage (BAL) cells 24 h after treatment with 2,2,6,6-tetramethyl-piperidin-1-oxyl (TEMPO) functionalized CNF [38], enzymatically pretreated CNF, bulk-sized pulp material [20], and carboxylated CNF [22]. In two studies, mice were sampled at 28 days post-exposure, and DNA damage was observed in the lungs or BAL cells of mice treated with enzymatically pretreated CNFs, carboxymethylated CNF, and bulk-sized pulp [20, 22].

In contrast to the in vivo experiments, earlier studies on the in vitro genotoxicity of CNFs have given negative results for unmodified, enzymatically pretreated, carboxymethylated, carboxylated, phosphorylated, sulfoethylated, hydroxypropyltrimethylammonium-substituted CNFs, as well as bulk-sized pulp for the induction of DNA or chromosome damage in human bronchial epithelial BEAS-2B cells [20, 36]. These findings have indicated that the cellulosic materials studied do not have primary genotoxic potential, and that the DNA damage observed in vivo is due to a secondary mechanism (possibly linked to inflammation) not present in traditional cell cultures of a single cell line.

In the present study, we have further characterized the possible genotoxic hazard of CNF. Birch fibres of four different surface compositions—unmodified, two anionic (TEMPO and carboxymethyl) and cationic (epoxypropyltrimethylammonium chloride)—obtained from the same source pulp, were fibrillated following the same procedure. For simplicity, we refer to the obtained fibrillated celluloses as cellulose nanofibrils (CNF), even though they also contained larger than nanosized fibres. Each type of these CNFs was size fractionated into three different fractions, with different relative axial aspect ratio. We examined a total of twelve different CNF samples, varying in surface composition and size, in human bronchial epithelial BEAS-2B cells, to assess the effect of both parameters as modulators of the formation of reactive oxygen species (ROS) and the potential induction of genotoxic effects by the CNFs. In addition, the non-nanosized source fibre was also evaluated to allow comparisons with the nanofibrils.

## Results

### Characterization of the cellulosic materials

Additional file 1: Fig. S1 presents scanning electron microscopy images of CNF samples at low magnification to show the structural organization of the nanofibrils. The morphology of the fibrils was quite heterogeneous, the surface modified fibrils being better fibrillated than the unmodified CNF. Furthermore, fibril aggregation was observed in all cases, which was expected from the fact that water was removed in the sample preparation.

**Fig. 1:**
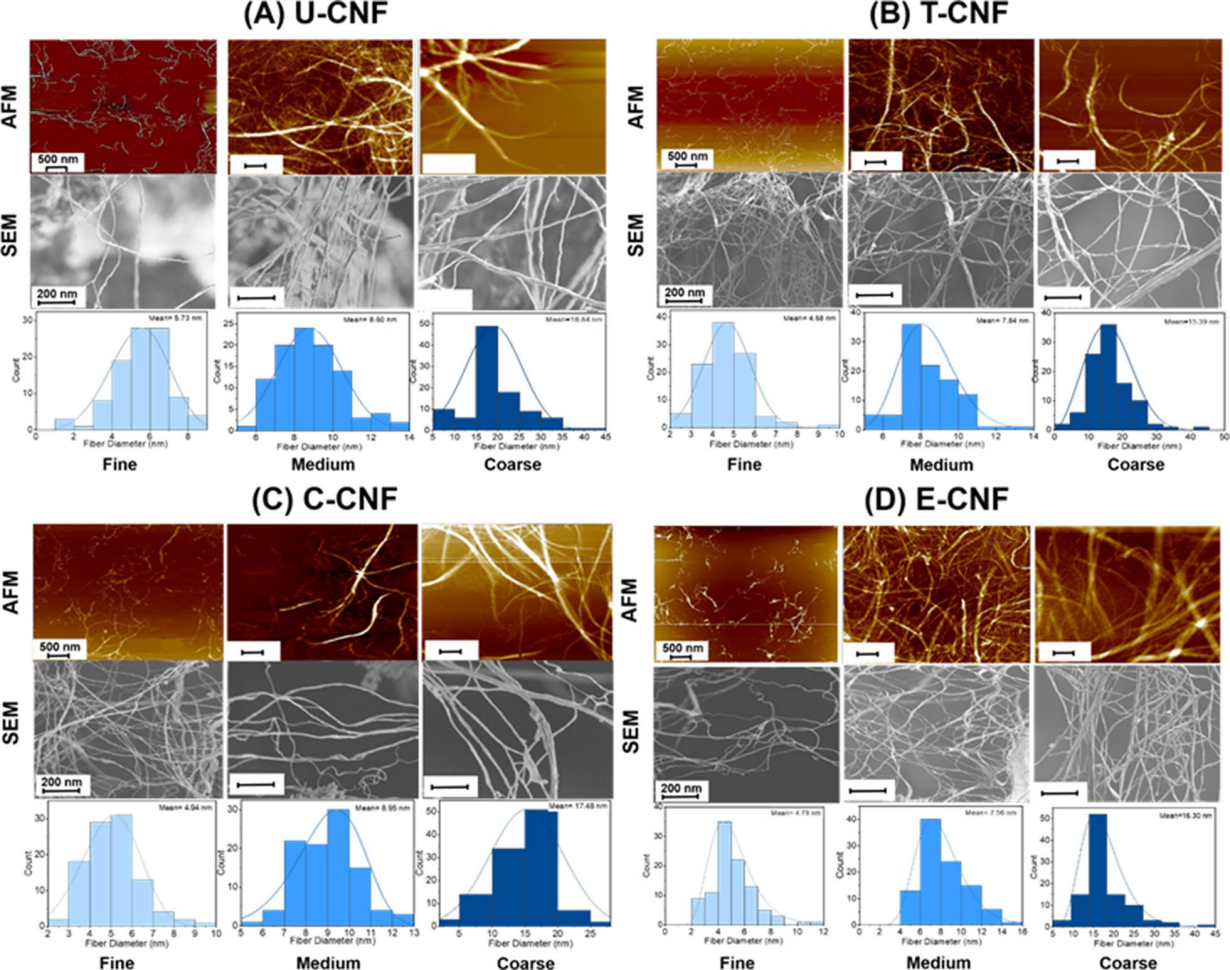
Atomic force microscopy (AFM) and scanning electron microscopy (SEM) images, and fibril diameter distributions (fitted log-normal models) of the three size fractions of U-CNF (**A**), T-CNF (**B**), C-CNF (**C**) and E-CNF (**D**) (N = 100 independent observations)

The structural morphology and fibre diameter distributions of the birch pulp fibres and the 12 CNF samples are shown in Additional file 1: Fig. S2 and Fig. 1, respectively. The calculated average diameter and length of individual nanofibrils, as well as the aspect ratio, are shown in Table 1. The lowest diameter and length among the nanofibrils were observed for the T-CNF samples, with values in the range of 4.7 ± 0.9 and 892 ± 301 nm for the fine, 7.8 ± 0.3 and 1491 ± 125 nm for the medium and 15.4 ± 0.2 and 2168 ± 352 nm for the coarse fractions, respectively. For all CNF samples, differences among the fine and coarse fractions were about 3- and 3.5-fold for diameter, and about 2- and 2.8-fold for length. The aspect ratio of the nanofibrils varied from 122:1 (coarse fraction of E-CNF) to 228:1 (fine fraction of C-CNF), the coarse fractions showing the lowest values. On the other hand, within each size fraction, T-CNF and E-CNF ranked as being the smallest in lateral size (diameter), whereas the C-CNF samples consistently showed a higher fibril length than the other samples. Consequently, the C-CNF samples generally had the highest axial aspect ratio. However, the observed differences among nanofibrils of different surface chemistry were small. All the nanofibrils showed a good degree of dispersion or homogeneity.

**Table 1:** Physico-chemical characteristics of the birch pulp fibres and fractionated fibrillated cellulose samples

Material			U-CNF	T-CNF	C-CNF	E-CNF	Source fibres
*Surface modification*			None	TEMPO oxidation	Carboxy-methylation	EPTMAC quaternization	None
*Degree of crystallinity (%)*			75	67	71	70	76
*Fibril dimensions (nm)*							
Fine		D	5.7 ± 0.1	4.7 ± 0.9	4.9 ± 0.1	4.8 ± 0.1	D: 16.2 ± 1.3 µm
		L	989 ± 256	892 ± 301	1127 ± 231	1020 ± 505	
		L/D	173	191	228	213	
Medium		D	8.6 ± 0.1	7.8 ± 0.3	8.9 ± 0.2	7.6 ± 0.3	L: 0.9 ± 0.1 mm
		L	1690 ± 640	1491 ± 125	1920 ± 321	1702 ± 124	
		L/D	197	190	215	225	
Coarse		D	18.8 ± 1.4	15.4 ± 0.2	17.5 ± 0.1	16.30 ± 1.0	L/D: 55.5
		L	2768 ± 521	2168 ± 352	2692 ± 232	1990 ± 129	
		L/D	147	141	154	122	
*ζ-potential (mV)*							
Fine	Water		− 16.5 ± 3.8	− 36.5 ± 3.2	− 36.7 ± 1.0	10.6 ± 0.2	Water: − 13.5 ± 1.6
	LHC-9		− 7.2 ± 0.6	− 1.8 ± 0.1	− 14.2 ± 1.3	3.3 ± 0.5	
Medium	Water		− 22.2 ± 2.1	− 41.5 ± 2.7	− 37.7 ± 1.6	31.6 ± 0.8	LHC-9: − 10.5 ± 0.6
	LHC-9		− 11.3 ± 1.1	− 3.9 ± 0.8	− 16.1 ± 1.5	11.5 ± 0.9	
Coarse	Water		− 20.3 ± 2.8	− 20.0 ± 2.5	− 19.9 ± 1.1	35.5 ± 1.0	
	LHC-9		− 9.8 ± 0.8	− 2.5 ± 0.3	− 11.4 ± 1.0	12.8 ± 1.4	

Fibre diameter (D) and length (L), and ζ-potential values are expressed as mean ± sd (N = 100 independent observations)

Table 1 also shows the ζ-potential values and degree of crystallinity of the source fibres and the 12 CNF samples. The ζ-potential values of the fractionated CNFs confirmed that cationic groups were introduced by EPTMAC quaternization (E-CNF), whereas the other CNF samples (U-CNF, T-CNF and C-CNF) were anionic (Table 1). T-CNF and C-CNF showed, in general, the highest absolute values of ζ-potential (higher electrostatic charge) in water, which suggested a better colloidal stability in the aqueous suspension. This is best evaluated by light scattering and rheology assessment, but qualitative observation of the turbidity revealed no major differences within the given size fraction. For a given CNF type, and with only one exception (E-CNF), a maximum in ζ-potential was generally observed for the fibrils belonging to the medium fraction. The reasons for this observation are not clear at this point but may relate to the distribution of colloidal matter during the protocols used in the fractionation by centrifugation.

There was a clear decrease in the absolute ζ-potential values when the materials were dispersed in the cell culture medium (Table 1). The ζ-values of the anionic T-CNF and C-CNF were similar in water but dropped more strongly for T-CNF than for C-CNF when diluted in culture medium.

The minimum and maximum apparent degree of crystallinity of the CNFs varied over a narrow range (67 and 75% for T-CNF and U-CNF, respectively) relative to that of the precursor material (76%) (Additional file 1: Fig. S3). Compared with the source fibres, the largest difference of CNF crystallinity was 9%, which is not negligible, but probably caused by several factors affecting the structure of cellulose in the samples. In general, and considering the variations in the X-ray diffraction data, no significant differences in crystallinity could be highlighted for the different CNF samples.

Results from the endotoxin assay (Additional file 1: Table S1) showed that the birch pulp fibres and the CNF samples, except for the three size fractions of T-CNF and the fine fraction of U-CNF, had a low level of endotoxin contamination, below the 0.5 EU/ml limit value established by the US Food Drug Agency for inhalation studies [40]. The three size fractions of T-CNF showed a high endotoxin level that exceeded the detection limit of the kit (> 1.2 EU/mL).

### Cellular uptake

The percentage of cells showing calcofluor-stained material is presented in Additional file 1: Table S2. In addition, examples of calcofluor staining in cells treated with the CNFs are shown in Additional file 1: Fig. S4. The staining can distinguish CNF agglomerates, but not individual nanofibrils. The calcofluor staining was performed on cellulase pre-treated slides. Therefore, stained CNF that appeared associated with some cells possibly reflected partial or completed cellular internalization. However, it may also have been due to CNF material attached to the cell membrane that was not efficiently eliminated by the cellulase treatment. Nevertheless, most cells did not show calcofluor-stained material, and the possible CNF internalization only concerned about 2% of the cells. No statistically significant differences among the samples were observed. In some cases (Additional file 1: Fig. S4C, D), CNFs seemed to be partially internalized by the cells. No calcofluor-stained material could be found in the untreated cultures (Additional file 1: Fig. S4H).

**Fig. 2:**
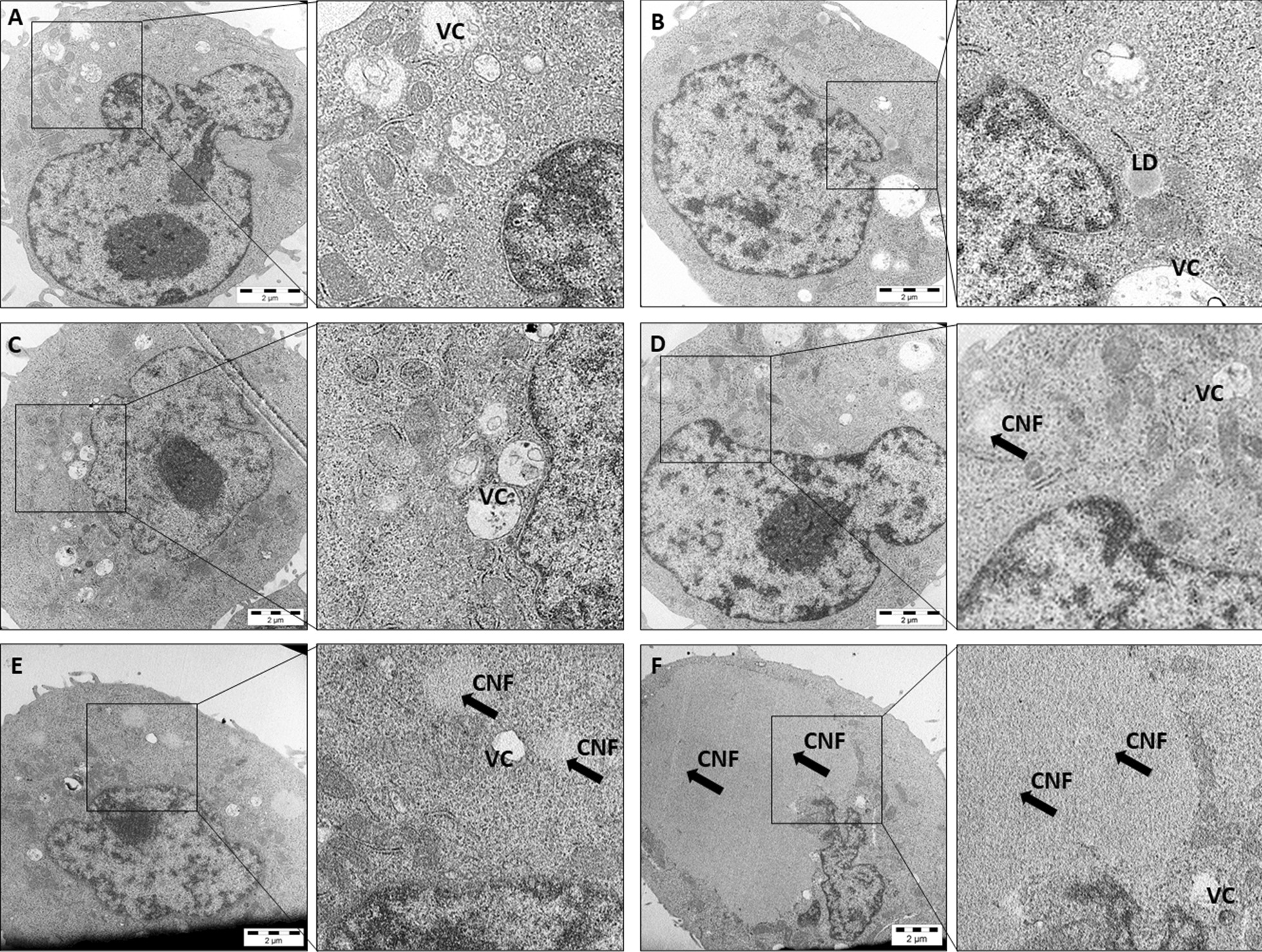
Transmission electron microscopic (TEM) images of BEAS-2B cells without treatment (**A**), and after a 6-h exposure to 111 µg/ml of coarse (**B**) and fine (**C**) U-CNF, fine C-CNF (**D**), and fine E-CNF (**E**, **F**). One cell shows a large mass of fine E-CNF inside the cytoplasm (**F**). CNFs are indicated by black arrows. VC: vacuoles; LD: lipid droplets (N = 15–20 independent observations)

To clarify whether CNFs were attached to the cell membrane or located within the cell, BEAS 2B cells treated with the CNFs were analyzed by transmission electron microscopy (TEM). As shown in Fig. 2, CNFs were visualized within the cytoplasm of some cells as vacuole-like structures. However, due to the low refractance of these materials, isolate internalized fibrils could not be distinguished.

### Cytotoxicity

Additional file 1: Fig. S5 summarizes results from the CellTiter-GloVR Luminescent Cell Viability Assay (expressed as percentages of living cells in comparison with the negative control cultures) after treating the cells with the birch pulp or the different types of CNFs for 24 or 48 h. The limit of 45 ± 5% cell viability (corresponding to the 55 ± 5% cytotoxicity established in OECD test guidelines) is indicated in each graph.

The results clearly illustrated that neither the pulp nor the CNFs were cytotoxic to BEAS-2B cells up to 1 mg/ml at the 24-h treatment. Conversely, the highest tested dose (1 mg/ml) decreased the number of living cells for all CNFs, but not for the pulp, after 48 h of treatment. The effect was the highest for C-CNF and E-CNF, regardless of the size fraction. However, as described below in the ‘chromosome damage’ sub-section, when cytotoxicity was assessed by the replication index (RI) in the cultures used for scoring the induction of micronuclei, the 55 ± 5% upper limit set by the guidelines was exceeded by none of the cellulosic materials, at none of the tested doses.

### ROS production

**Fig. 3:**
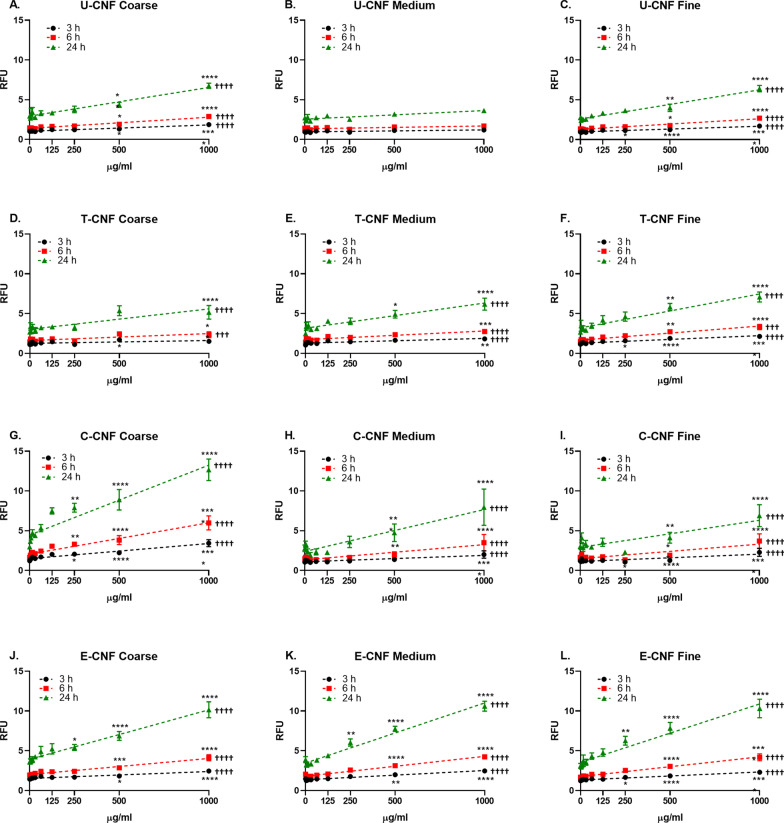
Cellular reactive oxygen species (ROS) production assessed by the fluorescent probe DCFDA in BEAS-2B cells after 3, 6 and 24-h exposure to coarse (**A**–**J**), medium (**B**–**K**) and fine (**C**–**L**) size fractions of U-CNF (**A**–**C**), T-CNF (**D**–**F**), C-CNF (**G**–**I**) and E-CNF (**J**–**L**). Data are expressed as relative fluorescence units (RFU) and presented as the mean ± se (N = 2 independent experiments). Asterisks designate statistically significant differences compared with the untreated cultures at *p < 0.05, **p < 0.01, ***p < 0.001 and ****p < 0.0001. Daggers designate statistically significant linear regression at ^†††^*p* < 0.001 and ^††††^*p* < 0.0001. The positive control, H_2_O_2_ (2 mM), induced a statistically significant increase in ROS production over the negative control values in all the experiments performed (7.9 ± 0.4-fold increase; *p* < 0.003) confirming the validity of the experiments (data not shown)

**Fig. 4:**
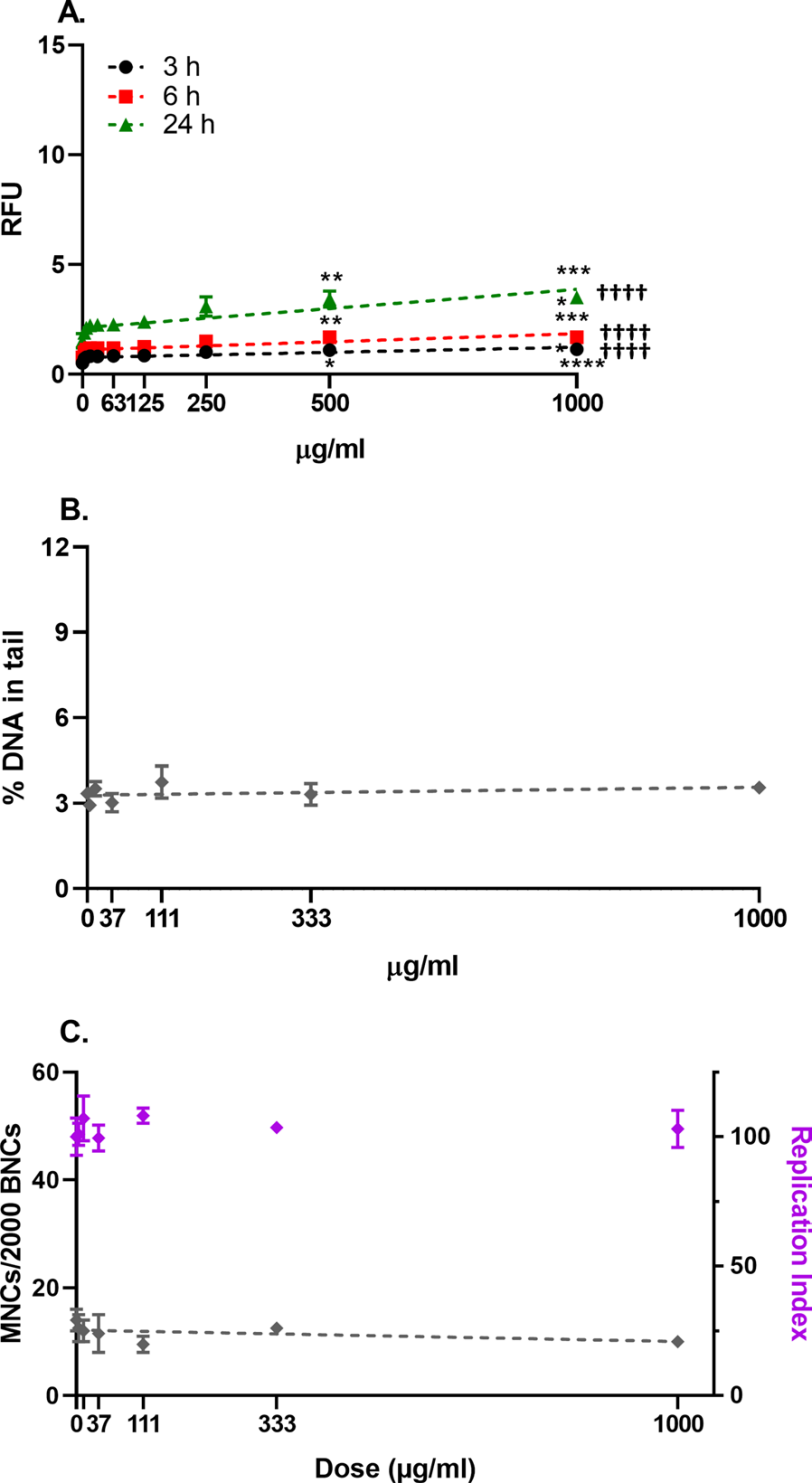
Cellular reactive oxygen species (ROS) production (**A**), DNA strand breaks (**B**) and micronucleus induction (**C**) in BEAS-2B cells after 3-h (**A**), 6-h (**A**), 24-h (**A**, **B**) and 48-h (**C**) exposure to pulp fibres. Data are presented as the mean ± se. Asterisks designate statistically significant differences compared with the untreated cultures at *p < 0.05, **p < 0.01, ***p < 0.001 and ****p < 0.0001. Daggers designate statistically significant linear regression at ^††††^*p* < 0.0001

Figure 3 shows the production of ROS by the different size fractions of the CNFs. The statistical analyses were performed separately for each size fraction (Table I of the’Analysis of covariance’ at the Additional file 1). ROS production by the pulp fibres is shown in Fig. 4A.

For the coarse fraction (Fig. 3A, D, G, J), there was a significant linear dose–response for the U-, C- and E-CNF samples at 3 h (*p* < 0.0001), but not for T-CNF. At later time points (6 and 24 h), a significant linear dose–response existed for all types of functionalization (*p* < 0.001), although the slopes of the regression lines (regression coefficients) differed in each case, the C-CNF sample showing the highest effectivity at all the time points. The highest dose of C-CNF induced a significant 2.8-fold increase in ROS formation in comparison with the untreated control already in the 3-h exposure (*p* < 0.0001), reaching up to a 4.3-fold increase after the 24-h exposure (*p* < 0.0001). E-CNF was the second most effective material, showing a 2.8-fold increase after 24-h (*p* < 0.0001). The C-CNF sample also showed the highest efficiency, with 250 µg/ml being the lowest dose statistically significantly different from the zero dose at 3-h (*p* < 0.05), 6-h (*p* < 0.01) and 24-h exposure (*p* < 0.01).

For the medium fraction (Fig. 3B, E, H, K), a significant linear dose–response (*p* < 0.0001) existed for T-CNF, C-CNF and E-CNF at the three time points, whereas no significant dose–response was found with U-CNF. The highest effectivity was shown by the E-CNF sample at all time points. The highest dose of E-CNF induced a significant 1.7-fold increase in ROS formation at 3-h (*p* < 0.0001), which raised up to a 2.7-fold increase after the 24-h exposure (*p* < 0.0001). The E-CNF sample also showed the highest efficiency, with 250 µg/ml being the lowest dose statistically significantly different from the zero dose at 24-h exposure (*p* < 0.01).

For the fine fraction (Fig. 3C, F, I, L), there was a significant linear dose–response (*p* < 0.001) for all types of functionalization at the three time points, and the slope of the regression lines was the same for all types of functionalization at 3 h, whereas the slopes differed at 6 and 24 h, E-CNF showing the highest effectivity. The highest dose of E-CNF induced a significant 2.5-fold increase in ROS formation at 6-h (*p* < 0.0001), and a significant 3.3-fold increase after the 24-h exposure (*p* < 0.0001). In addition, 250 µg/ml of E-CNF was the lowest dose statistically significantly different from the zero dose in the 3-h (*p* < 0.05) and 24-h exposure (*p* < 0.01).

Among the CNFs, the coarse U-CNF (Fig. 3A) was assumed to be the most appropriate to be compared with the original pulp fibres (Fig. 4A), as they only differed for the refining and microfluidization steps. Both cellulosic materials showed a significant linear dose–response (*p* < 0.0001) in the induction of ROS at the three time points (Table II of the’Analysis of covariance’ at the Additional file 1), the slope of the lines being similar at each time point, although the mean values of the nano-sized fibrils (U-CNF) were higher than those of the pulp.

### DNA damage (comet assay)

**Fig. 5:**
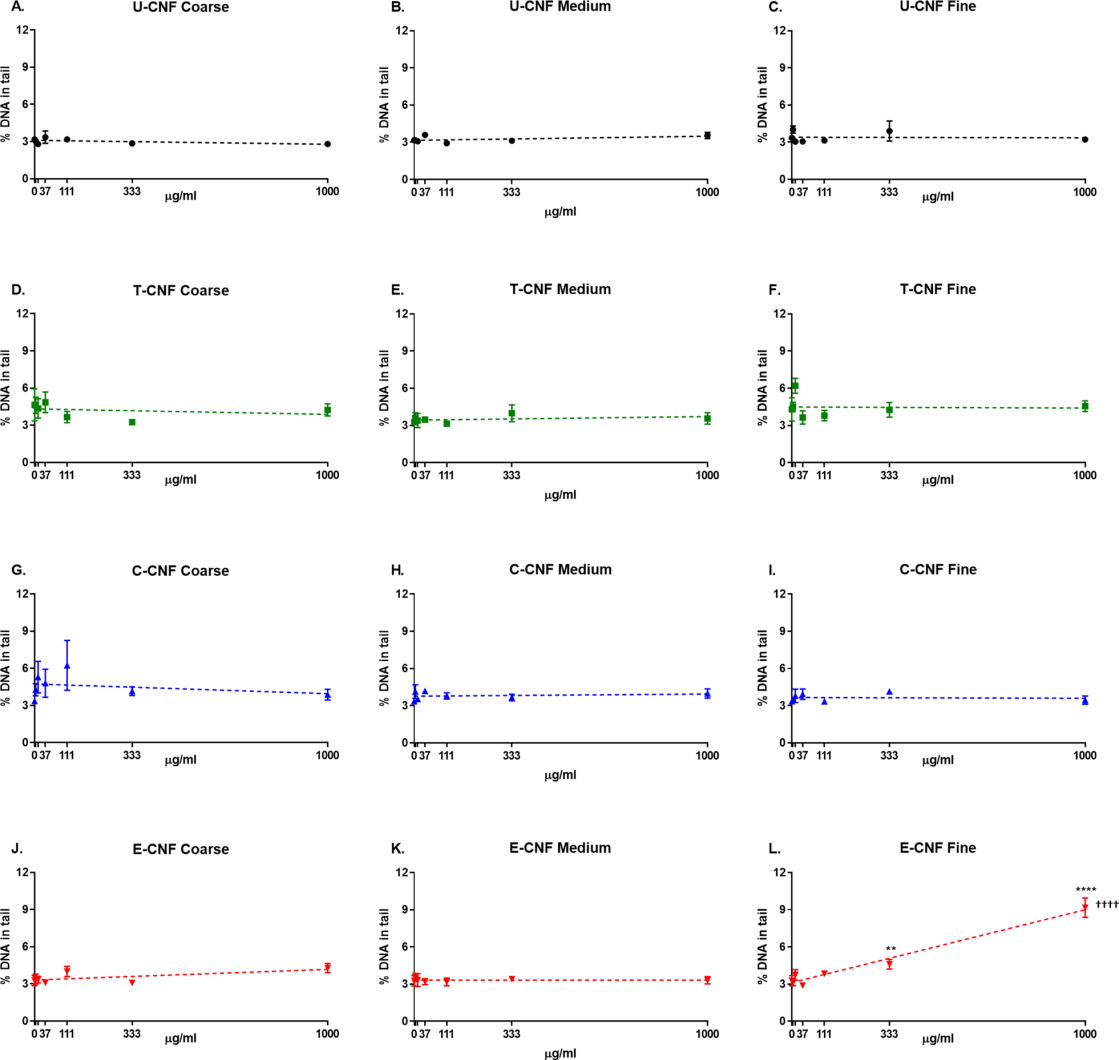
DNA strand breaks assessed by the comet assay in BEAS-2B cells after a 24-h exposure to coarse (**A**–**J**), medium (**B**–**K**) and fine (**C**–**L**) size fractions of U-CNF (**A**–**C**), T-CNF (**D**–**F**), C-CNF (**G**–**I**) and E-CNF (**J**–**L**). Data are expressed as percentage of DNA in tail and presented as the mean ± se (N = 2 independent experiments). Asterisks designate statistically significant differences compared with the untreated cultures at **p < 0.01 and ****p < 0.0001. Daggers designate statistically significant linear regression at ^††††^*p* < 0.0001. The positive control, H_2_O_2_ (20 mM), induced a statistically significant increase in the percentage of DNA in tail over the negative control values in all the experiments performed (15.2 ± 1.1-fold increase; *p* < 0.02) confirming the validity of the experiments (data not shown)

Figure 5 shows the induction of DNA damage by CNFs for each of the size fractions separately. No statistically significant linear dose-dependent increases or differences among doses were found for the coarse (Fig. 5A, D, G, J) and medium (Fig. 5B, E, H, K) fractions of any CNF. For the fine fraction (Fig. 5C, F, I, L), there was a statistically significant linear dose–response only for E-CNF (*p* < 0.0001), but not for the other CNF types. In addition, the two highest doses of fine E-CNF (333 and 1000 µg/ml) were statistically significantly different from the zero dose (*p* = 0.0029 and *p* < 0.0001, respectively). No dose effects were found for the other types of CNF.

No effect of the dose on the induction of DNA damage was observed for the pulp fibres (Fig. 4B), in agreement with results obtained with the coarse fraction of U-CNF.

### Chromosome damage (micronucleus assay)

**Fig. 6:**
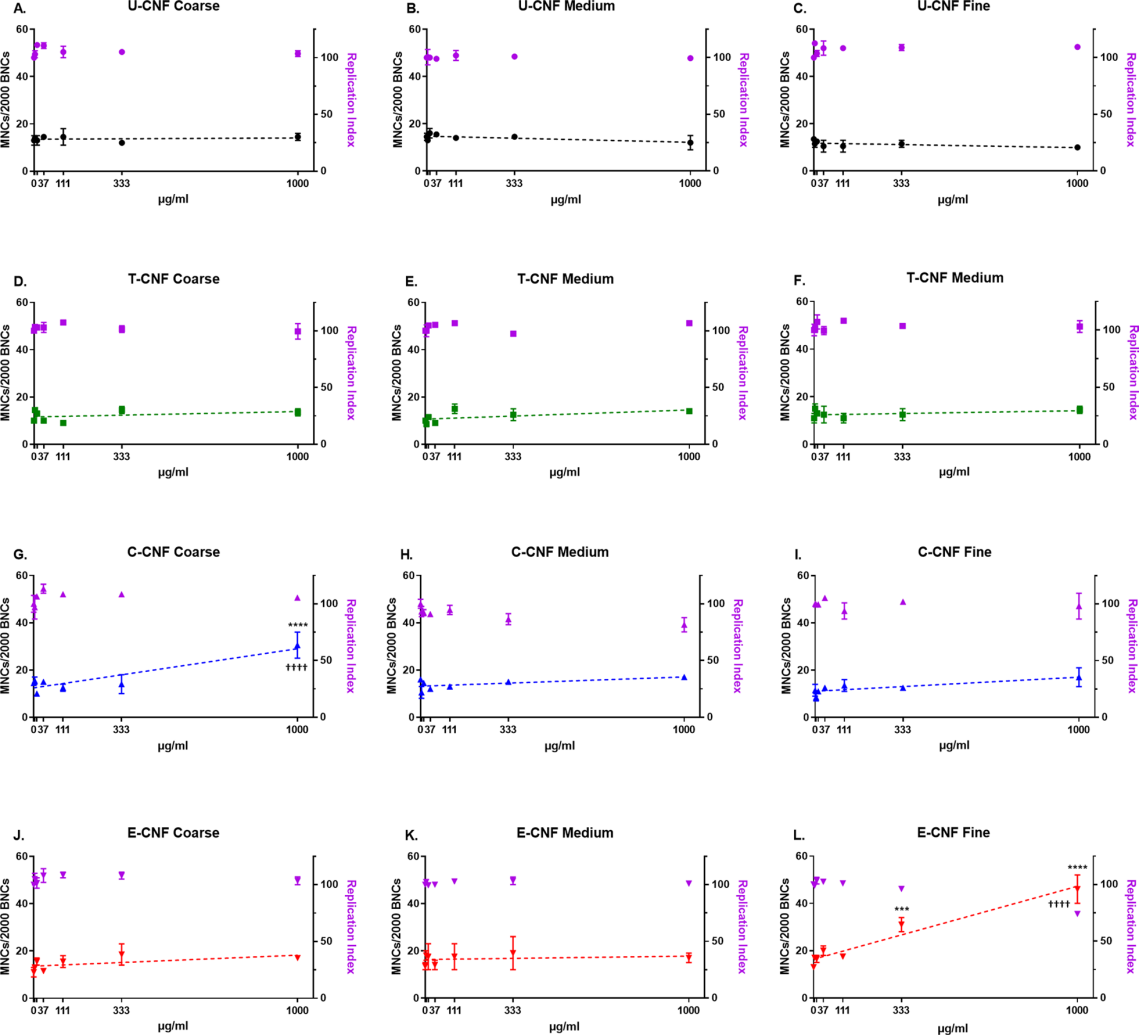
Frequency of micronucleated cells in 2000 binucleated cells (MNCs/2000 BNCs) and replication index (RI) after a 48-h exposure of BEAS-2B cells to coarse (**A**–**J**), medium (**B**–**K**) and fine (**C**–**L**) size fractions of U-CNF (**A**–**C**), T-CNF (**D**–**F**), C-CNF (**G**–**I**) and E-CNF (**J**–**L**). Data are presented as the mean ± se (N = 2 independent replicates). Asterisks designate statistically significant differences compared with the untreated cultures at ***p < 0.001 and ****p < 0.0001. Daggers designate statistically significant linear regression at ^††††^*p* < 0.0001. The positive control, MMC (150 ng/ml), induced a statistically significant increase in the frequency of micronucleated cells over the negative control values in all the experiments performed (21.59 ± 1.80-fold increase; *p* < 0.01; 27.6 ± 3.5% RI) confirming the validity of the experiments (data not shown)

Figure 6 shows the results of the induction of chromosome damage by CNFs for each of the size fractions separately.

Statistically significant linear dose-responses (*p* < 0.0001) only existed for C-CNF in the case of the coarse fraction (Fig. 6G), and for E-CNF in the case of the fine fraction (Fig. 6L). Besides, a statistically significant difference to the zero dose was observed for the two highest doses (333 and 1000 µg/ml) of fine E-CNF (*p* = 0.0005 and *p* < 0.0001, respectively) and the highest dose of coarse C-CNF (*p* < 0.0001). For the medium fraction, no significant linear dose-responses or differences among doses were observed.

No significant differences between the pulp fibres (Fig. 4C) and the coarse U-CNF, nor a dose-dependent effect or differences with the untreated cultures, were observed in the induction of chromosome damage.

Cytotoxicity, measured by the RI, was assessed in the same cultures that were used for scoring micronuclei. The results showed that a reduction in RI below 45 ± 5% of the concurrent negative control- corresponding to 55 ± 5% of cytotoxicity as set by the OECD guidelines [41] was induced by none of the cellulosic materials, at none of the tested doses (Figs. 4C, 6). The highest dose (1 mg/ml) reported values of 105.5 and 74.1% RI for coarse C-CNF and fine E-CNF, respectively; for all the other cellulosic materials tested RI was > 98%.

## Discussion

The present study aimed at investigating the effect of two material properties, size and surface composition, on the potential induction of genotoxic effects and ROS generation by CNFs when interacting with human lung cells. The selected human bronchial epithelial BEAS-2B cell line is considered to be a good model for human lung research [42]. BEAS-2B cells have a normal antioxidant capacity and are similar to primary bronchial cells as far as gene expression pattern [42, 43]. The non-tumorigenic origin of this cell line makes it suitable for genotoxicity studies, circumventing the problems that have been associated with chromosomal instability and increased oxidative stress response reported for some cancerous cell lines (e.g. A549) [44]. Furthermore, BEAS-2B cells can grow in serum-free medium, which allows testing the nanomaterials without the risks of serum-related modifications of their surface properties [45]. The BEAS-2B cell line has been extensively used in toxicological research on asbestos and nanomaterials [46].

### Characteristics of CNFs

Surface modifications are commonly introduced during the production of CNF to reduce the required energy of the process [4] and to ensure CNF with specific properties for different applications [29, 30]. The presence of surface charged groups in functionalized fibrillated cellulose samples results in a higher number of observed individual nanofibrils, as a consequence of electrostatic repulsion between the nanofibrils [29]. On the contrary, non-functionalized CNF is expected to present more nanofibril aggregates. Furthermore, differences in surface group density among the functionalized CNF materials influence the extent of fibre aggregation, with thicker fibres and more aggregates being observed for the less substituted CNFs [34]. Although the functional group content was not analyzed in the present study, a thorough TEMPO functionalization can be effective in achieving the maximum concentration of carboxylic groups on the surface of CNF. EPTMAC and the carboxymethyl treatments usually lead to lower degrees of substitution compared with TEMPO oxidation [6, 34]. Our results revealed that the nanofibrils produced by TEMPO mediated oxidation were quite homogeneous and displayed some of the smaller diameters compared with other fibrils. In fact, it has been reported that TEMPO oxidation is effective in individualizing cellulose nanofibrils [47]. However, following the values of the ζ-potential for T-CNF dispersed in the culture medium, one can expect a low colloidal stability for the same fibril types, possibly leading to some degree of agglomeration. The decrease in the absolute ζ-potential values in culture medium was also observed for the other CNFs. This is in agreement with previous observations with fibrillated celluloses [34, 36] and other nanomaterials [48, 49], as a consequence of the interactions of the nanofibrils with the biomolecules present in the cell culture medium, which screens the charges and therefore electrostatic repulsion.

Most of the available information about the size of fibrillated celluloses refers to fibril width as the length of CNF, which is commonly reported to be higher than 1 µm, is difficult to determine using microscopy techniques [4, 8]. In the present study, the length of individual nanofibrils (as well as pulp fibres) was estimated by measuring 100 fibres/nanofibrils per sample. In general, the results indicated that surface modification did not significantly affect the size of individual nanofibrils, within each size fraction. On the other hand, within a given surface modification type, the ranking of differences among the different fractions followed the order of diameter, followed by length and, to a lower extent, aspect ratio.

### Effect of size and surface chemistry on ROS generation and genotoxicity

In the present study, we found that all CNFs materials studied, except medium U-CNF, were able to induce a dose-dependent generation of intracellular ROS in BEAS-2B cells. ROS was also produced by the source pulp, similarly to coarse unmodified CNF. Two of the CNFs, the cationic fine E-CNF and the anionic coarse C-CNF, were capable of increasing genotoxic effects. Both caused a dose-dependent increase in the frequency of micronucleated cells, and fine E-CNF also induced a dose-dependent increase in DNA damage. Thus, our findings indicate that these two CNFs have primary genotoxic potential.

The mechanisms by which CNF could induce primary DNA damage in cells could either be direct—by direct interaction with the DNA molecule or its associated proteins—or indirect—by interfering with DNA replication or repair, or through the induction of oxidative stress or lipid peroxidation [50–52]. Direct interaction with the DNA would require the nanofibres to be taken up by the cells and enter the nucleus. On the other hand, direct interaction could also be possible during the mitosis when the nuclear membrane disappears [51]. Although primary direct genotoxicity has hardly been shown for poorly soluble nanomaterials in mammalian cells [51, 52], direct reaction with the DNA molecule has been reported in some cases [53].

In the present study, most cells showed no associated calcofluor-stained CNF, suggesting that CNF internalization only concerned a minority of the cells. Similar results were obtained in our previous study in BEAS-2B cells when using the same staining [36]. However, the fluorescence microscopic detection of calcofluor-stained CNFs does not allow distinguishing individual nanofibrils. Although we confirmed cellular uptake of CNFs by TEM analyses, the small number of cells that internalized CNFs, together with the difficulties to identify CNFs inside the cells [8, 54], precluded a quantitative comparison among the different types of CNFs using this technique. Neither was it possible to establish whether CNFs could enter the nucleus and interact with the DNA. In a recent study, CNFs showed adsorption on the plasma membrane of Kupffer cells and hepatocytes with limited cellular uptake, whereas cellulose nanocrystals were effectively taken up [55]. The nanocelluloses were labelled with fluorescent molecules, which may have interfered with their internalization.

Genotoxic nanomaterials are mostly assumed to act through an indirect mechanism, mainly by triggering the formation of ROS, which may cause oxidative DNA damage through free radical attack [51, 56]. A dose-dependent intracellular formation of ROS was induced by all cellulosic materials tested in the present study, except for the medium fraction of U-CNF. ROS could be generated by the internalized CNF either due to their inherently reactive surface, their interference with cell components, or by inhibition of the cellular antioxidant mechanisms [50]. Although the CNF-induced acellular ROS formation was not tested in the present study, a previous study suggested that CNFs have oxidating capacity by themselves [19]. Two types of CNFs (TEMPO oxidized and mechanically homogenized; 1000 µg/ml) showed a higher generation of hydroxyl radicals than cellulose nanocrystals and microcrystals in an acellular system mimicking the oxidative burst of macrophage attack. The TEMPO oxidized CNF—which had the smallest hydrodynamic diameter and crystalline size—was the most reactive one*.* Interestingly, none of the cellulose materials induced intracellular ROS in macrophage-like mouse leukemia RAW 264.7 cells, probably due to the short treatment used (10 min). On the other hand, nanomaterials may damage mitochondria, affecting mitochondrial respiratory chain and function [57]. Mitochondrial dysfunction leads to the production of ROS, which can also cause lysosome destabilization and release of lysosomal iron, hydrolytic enzymes and cathepsin B to the cytosol. Cathepsin B release can further damage mitochondria, leading to increased ROS formation and loss of cellular homeostasis [4]. However, ROS production could also raise because of an incomplete internalization of the nanofibrils. Complete and partial internalization of unmodified CNF materials has previously been reported with monocyte-derived dendritic cells [58]. Partial internalization of CNF was also observed in the present study. Finally, ROS could also be generated by the reaction of the nanofibrils with the cell surface inducing, e.g., membrane lipid peroxidation [59]. The latter would not require internalization of the nanofibrils. In fact, Menas et al. [37] reported that two types of unmodified CNF (freeze-dried powder and 0.9% wt gel) were not internalized by human adenocarcinomic alveolar epithelial A549 cells, but mostly localized at the cell boundaries. However, both CNFs induced a dose-dependent decrease in glutathione and protein sulfhydryl levels, indicating oxidative stress.

Substances with indirect genotoxicity are assumed to have a threshold mechanism of action [60]. The production of ROS should reach some level to trigger a genotoxic effect. Although most of the CNF samples we tested generated ROS, the coarse fraction of C-CNF and the fine fraction of E-CNF were the only materials able to induce genotoxic effects. They also showed the most effective induction of ROS within the respective size fractions. Hence, it is plausible that a ROS-mediated mechanism is involved, but further studies e.g., using ROS scavengers, would be needed to confirm such hypothesis. On the other hand, no genotoxic effects were induced by the medium or coarse fractions of E-CNF, despite they generated ROS with similar potency as fine E-CNF. Hence, intracellular ROS production alone did not explain the observed effects. Due to the inherent surface reactivity, CNF could induce lipid peroxidation, whose by-products could react with and damage the DNA molecule [61]. CNFs could also differ in their capacity to interact with proteins involved in cell division, and DNA replication, transcription or repair [50]. Furthermore, as the genotoxic fine and non-genotoxic medium and coarse fractions of E-CNF differed in length and diameter, nanofibrils size may have modulated the genotoxicity of E-CNF. A similar conclusion was reached by Manshian et al. [62] for the genotoxicity of single-walled carbon nanotubes (SWCNTs) of three different length ranges (400–800 nm, 1–3 µm and 5–30 µm) in BEAS-2B and MCL-5 human lymphoblastoid B cells. The intermediate length range showed the greatest level of ROS and expression of oxidative stress-related genes, and was the only SWCNT type that induced gene mutations. However, the induction of micronuclei inversely increased with SWCNT length, which suggested that oxidative stress alone did not explain the observed genotoxic profiles. In a recent study on nanocelluloses [55], the authors claimed to demonstrate a length-dependent mechanism of toxicity on liver cells in a cell type-dependent fashion. Short cellulose nanocrystals (149–715 nm), showing effective cellular uptake, triggered apoptotic cell death in Kupffer cells and hepatocytes, and mitochondrial ROS generation and pro-inflammatory cytokines in Kupffer cells. However, long CNFs (6.1–6.7 µm), which were not taken up by the cells, failed to induce significant toxic effects. However, besides length, these cellulosic materials were quite different also for other physico-chemical parameters (e.g. aspect-ratio or stiffness) that drive fibre toxicity.

In addition to length, diameter can also influence the toxicity of fibrous nanomaterials [24]. MWCNTs 50–80 nm in diameter induced a higher percentage of DNA damage in FE1-Muta(TM) mouse lung epithelial cells than two groups of smaller MWCNTs (diameter of 8–15 and 13–18 nm) [63]. Large MWCNT diameter was also associated with increased DNA damage in BAL fluid and lung tissue of mice [26]. In the present study, the coarse fraction of C-CNF induced genotoxic effects, unlike the other two size fractions of C-CNF, which had smaller diameters. On the other hand, the diameters of individual nanofibrils of the fine, medium and coarse E-CNFs were 4.8 ± 0.1, 7.6 ± 0.3 and 16.30 ± 1.0 nm, respectively. Assuming that this type of CNF could be internalized by the BEAS-2B cells, the finest nanofibrils might have more chances of entering the nucleus than the coarser ones, as the channel of the nuclear pores in human cells has a diameter of 5.2 nm [64]. Diffusion through the nuclear pores has been suggested as one of the mechanisms for nanomaterials entering the nucleus [56]. If so, fine E-CNFs could directly react with the DNA molecule or nuclear proteins, explaining the observed genotoxic effect.

We found DNA damage induction exclusively for fine E-CNF (cationic), while micronuclei were induced only by fine E-CNF and coarse C-CNF (anionic). Several studies have shown that surface charge may affect nanomaterial uptake and toxicity [24]. Nanomaterials with a positive surface charge have generally been reported to be more efficiently taken up by the cells and more toxic than those with a negative charge [65, 66]. Strongly cationic polyetherimide modified MWCNTs induced a high production of pro-fibrogenic cytokines and growth factors in BEAS-2B and THP-1 cells and a marked lung fibrosis in mice, whereas carboxylated MWCNTs did not have an effect [67]. In a study comparing poly(APMA) (cationic) and poly(NIPAAm) (anionic) -functionalized cellulose nanocrystals, the cationic material induced a stronger activation of NLRP3-inflammasome and increased the level of IL-1 β in murine macrophages, whereas the anionic material induced unspecific (NLRP3-dependent or independent) immunological effects [68]. Films of anionic (carboxymethylated) CNF showed higher cytocompatibility with human dermal fibroblasts than films of cationic (EPTMAC-quaternized) CNF [69]. Conversely, unmodified CNF induced a higher secretion of pro-inflammatory cytokines TNF-a and IL-1 β than carboxymethylated and EPTMAC-quaternized CNFs in THP-1 cells [34]. In another study of the same group, none of the tested CNFs, including anionic, cationic and unmodified samples, impaired the viability of Caco-2 cells [35]. However, the surface charge of the cationic samples included in both studies, measured by the ζ-potential values, changed in the presence of the culture medium, becoming negative. In the present study, E-CNF did not change its charge in the presence of culture medium but remained cationic. This was probably due to the lower protein content of the cell culture medium (LHC-9, not supplemented with bovine serum albumin) used in the present study. In fact, we observed the same result with another cationic CNF sample also tested in the same in vitro system and conditions [36]. Hence, the positively charged E-CNF samples may have been more efficiently taken up than the anionic CNFs, resulting in a higher intracellular ROS formation.

Among the anionic CNFs, the coarse C-CNF was the most potent inducer of ROS, followed by the medium C-CNF. In addition, the coarse C-CNF showed genotoxic effects. Based on the absolute ζ-potential values, the CNF samples showed slightly higher colloidal stability than the U-CNF samples. High dispersion stability was suggested to be associated with high internalization of MWCNTs in human adenocarcinoma alveolar basal epithelial A549 cells [70]. However, it is unclear how this parameter could affect the cellular uptake of CNF. On the other hand, C-CNF has a higher surface charge density of carboxyl groups than the unmodified CNFs [8]. The presence of carboxyl groups confers the nanofibrils with oxidizing properties [71]. However, the ROS induction potency of the C-CNF samples decreased with the size—and hence increasing surface area—of the nanofibrils.

As mentioned above, besides ROS formation, other mechanisms may have been involved in the induction of genotoxic effects by the coarse C-CNF and fine E-CNF. Interaction of nanomaterials with the mitotic spindle apparatus, centrioles or their associated proteins could cause aneugenic effects, which can also be detected by the micronucleus assay. For instance, aneugenic effects have been reported to be induced by MWCNTs [72] and graphene-based materials [71].

The other anionic CNFs assessed in the present study (T-CNF) were modified by TEMPO mediated oxidation, which introduces carboxylate groups (COOH) onto the CNF surface [30]. T-CNF has the highest surface change density of carboxyl groups among the analyzed CNFs. However, the T-CNF samples did not show higher ROS formation than other nanofibrils with lower density of carboxyl groups (e.g. U-CNF samples). On the other hand, the ζ-potential values of the T-CNF samples dropped dramatically when dispersed in the culture medium, which may have reduced the internalization of these nanofibrils by the cells. None of the size fractions of T-CNF showed genotoxic effects. Similarly, carboxylated few-layer graphene (FLG) did not increase the frequency of micronuclei in human lung epithelial 16HBE14o^−^ cells, unlike neutral—and amine functionalized-FLG [71]. On the other hand, introduction of COOH-groups onto the structure of one type of MWCNT suppressed the levels of oxidative DNA damage in A549 cells, although MWCNTs with high COOH content were able to induce DNA damage in the alkaline comet assay. However, COOH-modified MWCNTs induced a higher acellular ROS production than pristine MWCNTs [73].

The lack of induction of genotoxicity by T-CNF in the present study is in contrast with our previous studies in C57Bl/6 mice exposed by single (oro)pharyngeal aspiration (10–200 µg/mouse) to a TEMPO oxidised CNF (300–1000 nm in length, 10–25 nm in width), where increased DNA damage and an acute neutrophilic reaction were observed in the lungs at 24-h post-administration [38]. These results may suggest that TEMPO oxidized CNF operates by a secondary genotoxicity mechanism associated with inflammation, as has been reported for several other nanomaterials [56]. Such mechanisms cannot be assessed in the in vitro cultures of non-inflammatory cells [52, 56], but might be detected in cocultures of inflammatory and epithelial cells [52]. When A549 epithelial cells were cocultured with THP-1 macrophages, a TEMPO oxidized CNF (diameter 25.9 nm) was reported to induce a significant increase in micronuclei [74]. However, as both cell types in this coculture system were simultaneously treated with CNF (THP-1 cells apically and A549 cells basolaterally), it remained unclear if the observed genotoxicity was due to a primary or a secondary effect.

One surprising finding in the present study was the absence of ROS induction by the medium U-CNF, unlike all other CNFs. The medium U-CNF showed a higher ζ-potential value than the coarse and fine nanofibrils, indicating a higher colloidal stability. The same phenomenon was observed for the medium fractions of the other anionic CNFs which, however, were able to induce ROS formation. This exception is difficult to explain by typical analytical methods, but one can speculate that it is related to the confounding effects of chemical composition, which tracks with fibril width and residual components of the cell wall.

Our previous studies with the same cell model and assays as used in the present study did not show in vitro genotoxic effects for different surface functionalized CNFs [20, 36]. Aimonen et al. [36] reported no induction of DNA damage or micronuclei after studying a nonfunctionalized enzymatically pretreated (10–30 nm aggregates), carboxymethylated (10–15 nm aggregates), phosphorylated (4–5 nm fibrils), sulfoethylated (10–12 nm aggregates), or hydroxypropyltrimethylammonium-substituted (4–5 nm fibrils) CNFs, tested up to 500 µg/ml. However, increased generation of ROS was observed for the nonfunctionalized and the carboxymethylated CNFs (with the same surface chemistry as C-CNF), but not for the others, despite the hydroxypropyltrimethylammonium CNF had similar cationic surface chemistry as E-CNF. No induction of DNA damage or micronuclei was either observed by Lindberg et al. [20] for a carboxymethylated (length 5–50 µm; width 3–10 nm,), carboxylated (length 0.5–10 µm; width 4–10 nm,), and two enzymatically pre-treated (length 2–20 µm; width 2–15 nm and 7–20 nm, respectively) CNFs, and a bulk-sized pulp material (length 7–20 µm; width 30 µm,), when applying a dose level similar to that applied in the present study. The CNFs examined in the three studies ([20, 36] and the present one) derived from different sources and synthesis procedures, and different dispersion methods were applied. The CNFs evaluated in the present study and by Lindberg et al. [20] were only diluted and mixed by vortexing, whereas the CNFs of the study of Aimonen et al. [36] were sonicated to obtain the dispersions. Sonication may affect the structural properties of the material [54], and thereby alter the way the material interacts with cells. On the other hand, both previous studies worked with sterilized materials, whereas no sterilization procedures were used in the present study, except for the T-CNF samples. Furthermore, samples of wood CNF always contain micrometer-sized residual fibres, depending on the pre-treatments and fibrillation conditions applied during production [4, 6], as well as residual components of the cell wall (e.g. hemicellulose and lignin) which can be bound to cellulose, are difficult to remove or even to quantify [75], and may modulate the toxic effects.

The CNFs included in the study of Lindberg et al. [20] were also assessed in vivo [20–22]. The enzymatically pre-treated CNFs were more prone to trigger inflammation and induced increased DNA damage in the lung than those modified by carboxymethylation or carboxylation. These findings again suggest an involvement of secondary mechanisms of genotoxicity, mediated by inflammation. As pointed out by the authors, the inflammatory response in mice could have been induced by the presence of endotoxins in the CNF samples [20, 21]. Endotoxins have also been suggested to trigger the formation of intracellular ROS [36]. However, endotoxin contamination did not explain the genotoxic effects observed in the present in vitro study, as the formation of ROS did not correlate with a higher endotoxin level.

In considering the biological significance of the in vitro genotoxicity of coarse C-CNF and fine E-CNF, it has to be kept in mind that, although both materials showed a significant dose–response, only the highest doses (333 and 1000 µg/ml for fine E-CNF and 1000 µg/ml for coarse C-CNF) significantly differed from the untreated cultures, and that the highest dose was cytotoxic in the luminometric assay at 48-h exposure. However, no cytotoxic effects were reported by the RI. The discrepancy on the cytotoxicity results may reflect the different principle of the luminometric assay (depicting the absolute number of metabolically active cells) and the replication index (indicating the relative number of cell cycles per cell in treated cultures compared with control cultures), or the potential interaction of CNF (if remaining attached to the cells after washing) with the luciferase reagent preventing it to enter cells. The RI was measured in the same experimental cultures as the micronucleus assay and is, in fact, the recommended method to assess cytotoxicity [41]. Furthermore, no correlation between the cytotoxicity results of the luminometric method and the induction of genotoxic effects existed for the other size fractions of the same modified CNF samples.

### Comparison with the source cellulose fibres

Few studies have compared the source fibres with the CNF samples synthetized from them [18]. In the present study, pulp fibres were compared with the coarse fraction of U-CNF. Both materials induced a significant increase in the formation of ROS at the three time points assessed, but none of the materials induced genotoxic effects.

Pulp fibres (10,000 µm in length, 30 µm in width) and non-functionalized enzymatically pre-treated CNF (2–20 µm in length, 2–15 nm in width) did not activate macrophages in vitro [21] or induce genotoxic effects in BEAS-2B cells [20]. The materials induced a mild and acute inflammatory response, respectively, in mice 24 h after a single pharyngeal administration, which was however resolved after 28 days [21]. Treatment of mice with pulp fibres increased DNA damage in bronchoalveolar lavage cells at 24 h and in lung cells at 28 days following the exposure, whereas the unmodified CNF only induced an increase in DNA damage in lung cells 28 days after the administration [20].

## Conclusions

Our findings suggest cross-effects of surface chemistry and size as far as in vitro induction of intracellular ROS and genotoxic damage by fibrillated celluloses. One cationic (fine fraction of EPTMAC-functionalized) and one anionic (coarse fraction of carboxymethylated) CNF, which were the most effective producers of ROS in their corresponding size fractions, also induced genotoxic effects. A suggested primary genotoxic mechanism of action is through ROS generation, even though other mechanisms may also be involved. Although not genotoxic, the bulk source fibres also produced ROS, in a similar manner as unmodified CNF. In judging these findings, it has to be kept in mind that the CNFs showed very low cytotoxicity and that the genotoxic effects were observed at high doses. The conclusions cannot be generalized to all types of CNFs, as the synthesis process and the dispersion method used for testing affect their physico-chemical properties and, hence, their genotoxic effects. Therefore, further investigations are needed to better understand the mechanisms involved in the toxicity of CNF and the possible connections with their physico-chemical properties.

## Methods

### Synthesis and surface modification of CNFs

Nanomaterials can be contaminated by microbial components, such as bacterial endotoxin, which can trigger toxicological responses when cells and animals are exposed to them [76, 77]. Contamination with endotoxins can occur at any step of the manufacturing and handling process since most nanomaterials are produced in the absence of aseptic conditions [77]. In the case of cellulosic materials, most often occurring as aqueous suspensions, endotoxin contamination may become critical, as bacteria easily grow in cellulose-rich environments [78]. Consequently, cellulosic materials are usually sterilized (e.g., autoclaved) before testing [34–36].

We aimed at testing the fibrillated celluloses as produced, without further treatments (such as sterilization) that may modify CNF properties. Although the production processes occurred in a non-sterile pilot-scale laboratory, special care was taken to remove endotoxins and prevent bacteria contamination as much as possible. The surface of all the equipment used in the production and fractionation of the materials was extensively cleaned through consecutive washing with deionized water, ethanol (Altia, Helsinki, Finland), sterile purified water, and endotoxin-free water (Thermo Fisher Scientific, UT, USA). Sterile centrifuge tubes (Thermo Fisher Scientific, UT, USA) and sterile and endotoxin-free water were also used for preparing the samples in aqueous medium.

CNFs were sourced as a commercial, low endotoxin bleached sulfite birch wood fibres of dissolving-grade quality (UPM Kymmene Oyj, Finland), with a lignin content below 0.5%. The fibre suspension (pulp) was refined using a laboratory-scale PFI refiner (Hamjern Maskin AB, Norway). The refining degree, as measured by the standard Schopper-Riegler (°SR) method, changed from 17.5°SR to 74.5°SR, indicating a high water-holding ability, which correlates with a high degree of internal and external fibrillation. The refined fibres were used in their unmodified form or treated by TEMPO-(2,2,6,6-tetramethyl-piperidin-1-oxyl) oxidation, carboxymethylation, or epoxypropyl trimethylammonium chloride (EPTMAC) quaternization, as explained below. The unmodified and modified fibres were diluted to 1.5% solids content, and nanofibrils were obtained by disintegrating the fibres through a microfluidizer using six passes (Microfuidics, M-110 P, Massachusetts, USA), as previously reported [79, 80]. In this way, four different types of CNF were obtained: unmodified CNF (U-CNF) and CNFs produced from TEMPO-oxidized (T-CNF), carboxymethylated (C-CNF), and EPTMAC quaternized (E-CNF) fibres. Finally, three different size fractions (arbitrarily called fine, medium, and coarse) of each CNF type were obtained by subsequent centrifugations, according to the procedure described by Toivonen et al. [81] and Imani et al. [79, 80]. The resulting aqueous suspensions were concentrated by slow evaporation in an oven until reaching a 1.0–1.5% concentration, which was considered adequate for toxicity testing.

The procedure used in the preparation of the TEMPO-oxidized fibres is presented in Imani et al. [79, 80] except that the water used here was endotoxin-free, referred therein as “water”, for simplicity. In brief, 2 g of birch fibres (base on dry weight) were treated with NaBr and TEMPO oxidation (1.0 and 0.1 mmol/g, respectively) after dispersion at 1% (w/v) solids content in 200 cm^3^ water. Following this procedure, the mixture was mechanically stirred at 700 min^−1^ using an Ultra Turrax homogenizer for 5 min, and 100 ml of 10% (w/w) NaClO was added to maintain the pH at 10–11. The reaction was quenched after 3 h by washing the fibres three times with water.

The methodology used for the preparation of the carboxymethylated fibres is described in detail by Im et al. [82]. Briefly, the disintegrated pulp fibres were solvent-exchanged to ethanol by washing 1 g of the pulp three times in 100 ml ethanol and concentrated to 18 wt% solids content with an intermediate filtering step. The fibres were then impregnated for 30 min with 0.96 mmol/g of monochloroacetic acid in 200 ml isopropanol at 35 °C. These fibres were then added in portions to a solution of 3.68 mmol/g of NaOH dissolved in a 300 ml mixture of isopropanol and methanol at a volume ratio of 4:1. Finally, the pre-treated fibres were washed with water and filtered until conductivity and pH reached set values of ≤ 20 µS/cm and 7.0 ± 0.5, respectively.

The preparation of the EPTMAC-quaternized fibres was performed according to the method detailed by Kono et al. [83]. In brief, 1 g of the pre-treated pulp was completely suspended in 1.5 M NaOH (25 ml) at 4 °C, and EPTMAC (1.4 g, 9.2 mmol) solution was then added dropwise into the suspension. After stirring in a hot water bath at 60 °C for 24 h, the reaction mixture was poured into acetone (100 ml). The precipitate was filtered and then neutralized with 1:1 aqueous acetone/water (200 ml) to remove unreacted EPTMAC.

In summary, twelve different CNF samples of four different surface chemistry (U-CNF, T-CNF, C-CNF, and E-CNF) and three size fractions (coarse, medium, and fine) were produced. The original wood fibres were also included in the toxicological analyses.

### Morphological and surface characterization of the fractionated CNFs

Atomic force microscopy (AFM) was applied to investigate the morphology of the CNF samples, using a Dimension 5000 scanning probe microscope (Veeco, TX, USA). The measurements were performed in tapping mode by a Veeco Nanoscope with V controller (Veeco) in air using MicroMash silicon cantilevers (NSC15/AIBS). The samples were prepared by dispersing the respective CNF (0.001 wt%) in an ultrasonic bath (240 W, 50/60 Hz; BANDELIN, Germany) for 30 min. A droplet of the sample was cast onto a microscope glass slide and allowed to dry at room temperature for 24 h.

Scanning electron microscopy (SEM) analyses were carried out using a field emission gun scanning electron microscopy (FEG-SEM) microscope (Zeiss Sigma VP, Jena, Germany) at an acceleration voltage of 1.5 kV with a field emission gun. The samples were freeze-dried (0.001 wt. %) overnight and gold-sputtered to a thickness of 5 nm. X-ray diffraction (Model X’Pert PRO, Philips PANalytical, Netherlands) spectra were carried out to determine the crystallinity of the fractioned CNFs from dried suspensions. The samples were scanned in the range of 2θ = 5 − 50° with a scanning rate of 0.5° min^−1^ at 45 kV and 40 mA. The images obtained from the AFM and SEM analyses were subjected to ImageJ (USA) analysis by using 100 nanofibrils from each size fraction, yielding the respective size distribution profile.

Bacterial contamination was tested using the 3 M™ Petrifilm™ Aerobic Count Plates. Bacterial lipopolysaccharide content (endotoxin levels) was measured using the Pierce™ Chromogenic Endotoxin Quant Kit (Thermo Fisher Scientific, Waltham, MA, USA), which has no interference from β-glucans, as previously described [77]. In brief, CNFs were dispersed at 100 μg/ml in endotoxin-free water and heated at 75 °C for 15 min to promote endotoxin release. The samples were then placed in duplicate (50 μl each) into a 96-well microplate, and the following reagents were added and incubated at 37 °C according to the specific instructions of the kit: LAL reagent (50 μl per well), substrate solution (1:1) solution (100 μl per well), and 25% acetic acid (100 μl) to stop the reaction. The microplates were analyzed within the following 30 min with a photometer reader at 405 nM. Endotoxin levels were determined by comparison to a standard curve (range 0.15–1.2 EU/ml) run in parallel within the same experiment. Possible interference with photometric measurement was excluded by using blank samples. Interference (inhibition or enhancement) was evaluated according to the kits’ instructions by spiking the samples with 0.6 EU/ml and calculating spike recovery percentage. A recovery of < 50% was considered significant inhibition, < 75% moderate inhibition, > 125% moderate enhancement and > 150% significant enhancement.

### Dispersion and characterization of the CNFs in the culture medium

Aqueous CNF suspensions (1.0–1.5%) were diluted to 2 mg/ml in LHC-9 medium (Gibco, Life Technologies Corporation, Grand Island, NY, USA). Stock dispersions were then mixed vigorously by high-speed vortexing for 20 s. Then, serial dilutions were prepared in culture media, and mixed with vortex for 20 s before being added to the cells. In the case of the T-CNF fractions, due to the presence of bacterial contamination, the stock solution was sterilized by autoclaving before further dilutions in the culture medium.

The electrostatic charge of the fibrils was assessed by using electrophoretic mobility, measured as ζ-potential. Dispersions of 0.001% (w/w) of the birch pulp fibres and CNF samples were prepared in water and in cell culture medium through ultrasonication for 60 s using a universal dip cell in a ZetaSizer Nano instrument (Nano ZS, Malvern Instruments Ltd, UK). The measurements were repeated three times for each sample at 25 °C.

### Cell culture

Transformed human bronchial epithelial BEAS-2B cells were obtained from the American Type Culture Collection through LGC Promochem AB (Borås, Sweden). The BEAS-2B cells were grown in serum-free LHC-9 medium (Gibco, Life Technologies Corporation, Grand Island, NY, USA) at 37 °C in a humidified atmosphere of 5% CO_2_. Log-phase BEAS-2B cells were plated on 48-well plates (comet assay), 96-well plates (cytotoxicity and reactive oxygen species), and 2-well chamber slides (micronucleus assay) (Nunc, Roskilde, Denmark) from one to three days prior to exposure to the cellulosic material samples.

### Cytotoxicity

The purpose of the cytotoxicity assays was to choose the range of CNFs and pulp doses to be tested in the genotoxicity assays. According to OECD guidelines on the in vitro micronucleus assay [41], the highest test substance concentration to be included in the assay (in the absence of micronucleus induction at earlier doses) should produce 55 ± 5% cytotoxicity. Higher levels may induce chromosome damage as a secondary effect of cytotoxicity [84] and should, therefore, be avoided.

To fulfil the guidelines’ requirements, and due to the previously reported low cytotoxicity displayed by CNFs and the limited cellular uptake, we needed to test up to 1000 µg/ml. Calculation of the actual cell concentrations was not possible, as the existing models (e.g. ISD3) do not seem appropriate for fibres. A technique for measuring the in vitro particle kinetics of CNF suspended in cell culture medium has recently been developed [54]. According to the authors, in the case of CNF, the administered mass equals the mass deposited on cells.

BEAS-2B cells were seeded in white clear flat bottom 96-well plates (Corning, Kennebunk, ME, USA) at a density of 2 × 10^4^ cells/well (200 µl/well; culture area 0.32 cm^2^/well) and grown to semiconfluency, after which they were exposed to the cellulosic material dispersions for 24 and 48 h at 9 doses: 4, 8, 16, 31, 63, 125, 250, 500 and 1000 µg/ml (equivalent to 2.4, 4.9, 9.8, 19.5, 39.1, 78.1, 156.2, 312.5 and 625.0 µg/cm^2^). 0.1% Triton X-100 (Applichem, Darmstadt, Germany) was used as a positive control, while untreated cells served as a negative control at each time point. All treatments were performed in quadruplicate, and the experiments were repeated twice.

Cytotoxicity was measured using the CellTiter-GloVR Luminescent Cell Viability Assay (Promega, Madison, USA) according to instructions provided by the manufacturer (Technical bulletin, Promega Corporation, revised 6/09, part# TB288), and as previously described [36]. In brief, after the treatment, the exposure medium was replaced with 100 µl of fresh culture medium per well. Then, the plates were incubated for 3.5 h at 37 °C and equilibrated at room temperature for 30 min. 100 µl of CellTiter-Glo^®^ One Solution were added per well, and the contents were mixed for 20 min on an orbital shaker to induce cell lysis. The plates were then incubated at room temperature for 10 min to stabilize luminescent signal, and luminescence was recorded using a plate reader (Fluoroskan Ascent FL, Vantaa, Finland). Optical interference with the materials was assessed in parallel wells without cells. Exposure medium was not replaced in these controls before adding the reagent. However, the cellulose materials showed no interference with the luminometric determinations.

Cytotoxicity was expressed as the relative luminescence in the treated cultures in comparison with the control cultures. This assay reflects all treatment-related effects (necrosis, cell cycle delay, and apoptosis) that reduce the number of viable or living cells. In addition, as requested by the OECD TG 487 [41], the replication index was always measured when performing the micronucleus assay. In this way, the adequacy of the chosen dose range based on the luminometric assay could be checked.

### Reactive oxygen species production

The levels of intracellular reactive oxygen species (ROS) were measured using the chloromethyl derivative of 2′,7′-dichlorodihydrofluorescein diacetate (CM-H_2_DCFDA) (Invitrogen, Eugene, OR, USA) according to the manufacturer’s guidelines.

BEAS-2B cells were plated on black clear flat bottom 96-well plates (Corning, NY, USA) at a density of 20,000 cells/well (200 µl/well; culture area 0.32 cm^2^/well) and grown to semiconfluency for two days. After being washed with Dulbecco's phosphate-buffered saline (PBS; Biowest, Nuaillé, France), the cells were loaded with 2.5 µM CM-DCFDA in PBS for 30 min at 37 °C. Thereafter, the loading buffer was removed, and the cells were washed with PBS. Then the cells were treated with the cellulosic material dispersions at 9 doses: 4, 8, 16, 31, 63, 125, 250, 500 and 1000 µg/ml (equivalent to 2.4, 4.9, 9.8, 19.5, 39.1, 78.1, 156.2, 312.5 and 625.0 µg/cm^2^). 2 mM H_2_O_2_ (Sigma-Aldrich Chemie, Steinheim, Germany) was used as a positive control, while untreated cells served as a negative control at each time point. Fluorescence was recorded at 3, 6 and 24 h (excitation 485 nm, emission 538 nm) using a plate reader (Fluoroskan Ascent FL, Vantaa, Finland). The average fluorescent intensity was calculated by subtracting background values*.* All treatments were performed in quadruplicate, and the experiments were repeated twice.

### Genotoxicity assessment

#### Comet assay

The comet (single cell gel electrophoresis) assay was used to study DNA strand breaks and alkaline labile sites in BEAS-2B cells after exposure to the cellulose materials. BEAS-2B cells in log phase were plated in 48-well plates (culture area 0.95 cm^2^/well, culture medium volume 250 µl/well; Corning, NY, USA) two days prior to exposure. Exposure time was 24 h, and the cells were exposed to 4, 12, 37, 111, 333, 1000 µg/ml (corresponding to 1.0, 3.1, 9.7, 29.2, 87.6 and 263.1 µg/cm^2^) of each cellulose material. Untreated controls and positive controls treated with hydrogen peroxide (20 mM, Riedel–de Haen, Seelze, Germany) were included in all series. All treatments were performed in duplicate, and the experiments were repeated twice. The comet assay was performed in alkaline conditions (pH > 13) as described previously [85]. The slides were coded, and one scorer performed the comet analysis using a fluorescence microscope (Axioplan 2, Zeiss, Jena, Germany) and an interactive automated comet counter (Komet 5.5, Kinetic Imaging Ltd., Liverpool, UK). The percentage of DNA in the comet tail from 200 cells per dose and experiment (two replicates per dose, two slides per replicate, 50 cells/slide) was used as a measure of the amount of DNA damage.

#### Cytokinesis-block micronucleus assay

The cytokinesis-block micronucleus (CBMN) assay was applied to study chromosomal damage in BEAS-2B cells after exposure to the cellulose material samples. The cells were plated on 2-well chamber slides (culture area 4.2 cm^2^/well, culture medium volume 2 ml/well) at a density of 150,000 cells per well and incubated for 48 h, to reach semi-confluence, prior to the treatment.

Based on the cytotoxicity assay, the cells were exposed for 48 h to six doses of the dispersed materials: 4, 12, 37, 111, 333, and 1000 µg/ml (corresponding to 1.9, 5.7, 17.6, 52.9, 158.6, 476.2 µg/cm^2^). Cytochalasin B (9 µg/ml; Sigma–Aldrich Chemie, Steinheim, Germany) was added to the cell cultures 6 h after starting the treatment, to induce binucleation of dividing cells. Untreated cultures and cultures treated with the positive control mitomycin C (MMC; Sigma–Aldrich, Steinheim, Germany; 150 ng/ml) were included in all experiments. All cultures were prepared in duplicate. After the exposure, the cells were processed and stained as previously described [36].

The slides were coded, and the frequency of micronucleated cells in 4000 binucleate cells per dose (2000 cells/replicate) was analyzed by one scorer using a ZEISS Axio Imager Z1 microscope (Carl Zeiss AG, Oberkochen, Germany). Binucleate cells and micronuclei in them were identified using a 40 × objective lens using a FITC/TRITC double filter for acridine orange, and micronuclei were verified with DAPI. The replication index (RI), one of the recommended measures of cytotoxicity [41], was determined from 200 cells per dose (100 cells/replicate) as follows:

$${\text{RI}} = \frac{{\left( {{\text{No}}.\;{\text{binucleated}}\;{\text{cells}} + 2*{\text{No}}.\;{\text{multinucleated}}\;{\text{cells}}} \right)/{\text{Total}}\;{\text{no}}.\;{\text{of}}\;{\text{cells}}\;{\text{treated}}\;{\text{cultures}}}}{{\left( {{\text{No}}.\;{\text{binucleated}}\;{\text{cells}} + 2*{\text{No}}.\;{\text{multinucleated}}\;{\text{cells}}} \right)/{\text{Total}}\;{\text{no}}.\;{\text{of}}\;{\text{cells}}\;{\text{control}}\;{\text{cultures}}}}$$

#### Cellular uptake of fibrillated celluloses

To assess the potential internalization of CNF by BEAS-2B cells, CNF was stained with the Calcofluor White Stain (Merck KGaA, Darmstadt, Germany) as previously described [36], based on the method described by Tomić et al. [58]. The staining was performed on the same slides that were used for the scoring the micronucleus frequency (for slide preparation, see above) from cultures treated with 111 µg/ml of the coarse and fine fractions of the CNF samples and the pulp fibres. At harvest, the cells were treated for 15 min at 25 °C with cellulase (8.7 µl/ml, Cellic CTec2, Novozymes, Bagesvaerd, Denmark) to remove excess CNF outside the cells. A drop of calcofluor was applied onto acridine orange-stained slides and incubated under a cover slip for 1 min. The sample was thereafter examined with a fluorescence microscope (ZEISS Axio Imager Z1, Carl Zeiss AG, Oberkochen, Germany), using DAPI/FITC/TRITC triple filter and 40× objective lens. One thousand cells per sample (500 cells/replicate) were analyzed for the presence of calcofluor-stained CNF.

Further assessment of cellular internalization was done by transmission electron microscopy (TEM) analyses. BEAS-2B cells were plated on 24-well plates (Corning, NY, USA) at a density of 5 × 10^4^ cells/well (500 µl/well; culture area 1.9 cm^2^/well) and grown to semiconfluency, after which they were exposed to the coarse and fine fractions of the CNF dispersions for 6 h at 111 µg/ml (equivalent to 29.2 µg/cm^2^). Cell samples were processed for transmission electron microscopy (TEM) analyses as previously described [86]. About 15–20 cells per sample were analyzed using a Jeol JEM 1220 TEM (Tokyo, Japan) operated at an acceleration voltage of 100 kV and equipped with a TEM CCD camera and iTEM software (Veleta, Olympus Imaging Solutions GMBH, Münster, Germany).

### Statistics

Comparisons among the CNF samples, as well as between the pulp fibres and the coarse fraction of the U-CNF, for the induction of intracellular ROS, DNA damage, and chromosome damage were carried out by the analysis of covariance (ANCOVA), according to the models described in the Additional file 1 (Analysis of covariance). A Bonferroni pairwise comparison of means test was applied to assess whether, for each cellulosic material, any of the doses significantly differed from the corresponding zero dose. Comparison among CNF samples for the percentage of cells showing calcofluor-stained material was done by a two-way analysis of variance (ANOVA). Comparisons between the positive and negative control groups in the ROS, comet and micronucleus assays were performed by unpaired one-tailed t-test. The effects were considered significant if *p* < 0.05. The analyses were performed using the IBM SPSS Statistics for Windows (2013), Version 22.0 program.

## Supplementary Information

**Additional file 1.** Analysis of covariance (ANCOVA).

## Data Availability

The datasets used and analysed during the current study are available from the corresponding author on reasonable request.
