# Supplementary material for: Surface functionalization and size modulate the formation of reactive oxygen species and genotoxic effects of cellulose nanofibrils
Source: Part Fibre Toxicol. 2022 Mar 16;19:19. doi: 10.1186/s12989-022-00460-3 (PMC8925132; doi:10.1186/s12989-022-00460-3)
Supplement: Supplementary file 1 — Additional file 1. Analysis of covariance (ANCOVA). [file 12989_2022_460_MOESM1_ESM.pdf]

Supplementary material

Surface functionalization and size modulate the formation of reactive oxygen species and genotoxic effects of cellulose nanofibrils

Aimonen K[a], Imani M[b], Hartikainen M[a], Suhonen S[a], Vanhala, E[a], Moreno C[c], Rojas OJ[b,d], Norppa H[a] and Catalán J[a,c,*]

[a] *Finnish Institute of Occupational Health, Box 40, 00032 Työterveyslaitos, Helsinki, Finland*
[b] *Department of Bioproducts and Biosystems, Aalto University, Espoo, Finland*
[c] *Department of Anatomy, Embryology and Genetics, University of Zaragoza, Zaragoza, Spain*
[d] *Bioproducts Institute, Departments of Chemical and Biological Engineering, Chemistry and Wood Science, The University of British Columbia, Vancouver, British Columbia, Canada*

Analysis of covariance (ANCOVA)

A. Toxic responses elicited by the cellulose nanofibers

In this study, three toxicological endpoints were independently analyzed: ROS induction (at 3, 6 and 24 h of exposure), DNA damage and chromosome damage. These three endpoints were evaluated as dependent variables by a model composed of two factorial factors (categorical independent variables) and a covariate, where surface functionalization (F) was the first factor with four levels (U-CNF, T-CNF, C-CNF and E-CNF), size fraction (S) the second factor with three levels (coarse, medium and fine), and dose (D) a categorical covariate with ten levels for ROS induction, and seven levels for DNA and chromosome damage. The model was analyzed by an analysis of covariance (ANCOVA).

The results from ANCOVA revealed that there was a statistically significant three-way interaction among Functionalization (F), Size fraction (S) and Dose (D) for all the toxicological endpoints analyzed ($p < 0.0001$). This means that none of these factors always induced the same effect. Instead, they influenced one another.

One way of analyzing the three-way interaction is using tests of simple main effects. That is, the effect of a set of variables across the levels of another variable. In this case, we examined the Functionalization*Dose interaction (F*D) for each of the levels of size (S), which is the factor with the lower number of levels, as shown in Table I.

Table I Probability (p-value) of the Functionalization*Dose interaction for each level of size in each toxicological endpoint

Endpoint	Size fraction		
	Coarse	Medium	Fine
**ROS induction**			
3 h	$p < 0.0001$	$p = 0.0072$	NS
6 h	$p < 0.0001$	$p < 0.0001$	$p = 0.0045$
24 h	$p < 0.0001$	$p < 0.0001$	$p < 0.0001$
**DNA damage**	NS	NS	$p < 0.0001$
**Chromosome damage**	$p < 0.0001$	NS	$p < 0.0001$

NS: non-significant ($p > 0.05$)

ROS induction (3 h)

The significant three-way interaction among F*D*S was justified by the significant interaction between Functionalization and Dose (F*D) that existed for the coarse and medium size fractions, but not for the fine one (Table I). For the coarse fraction, the significant F*D interaction was justified by the existence of a significant linear dose-response for U-CNF, C-CNF and E-CNF, according to the following models:

U-CNF: $Y_i = 1.039 + 0.0008\, X_i$ $p < 0.0001$

C-CNF: $Y_i = 1.490 + 0.0019\, X_i$ $p < 0.0001$

E-CNF: $Y_i = 1.529 + 0.0009\, X_i$ $p < 0.0001$

No significant linear dose-response existed for T-CNF.

A Bonferroni pairwise comparison of means test was applied to assess whether, for each type of functionalization, any of the doses significantly differ from the corresponding zero dose. The highest two doses of U-CNF (500 and 1000 µg/ml; $p = 0.0217$ and $p < 0.0001$,

respectively), the three highest doses of C-CNF (250, 500 and 1000 µg/ml; $p = 0.0110$, $p < 0.0001$ and $p < 0.0001$, respectively), and the two highest doses of E-CNF (500 and 1000 µg/ml; $p = 0.0129$ and $p < 0.0001$, respectively) were significantly different than the corresponding untreated controls. For T-CNF, the dose of 500 µg/ml also significantly differed from the corresponding zero dose ($p = 0.0011$).

On the other hand, in the case of the medium fraction, the significant interaction detected between Functionalization and Dose (F*D) was justified by the existence of a significant linear dose-response for T-CNF, C-CNF and E-CNF, whereas non-significant dose-response was found with U-CNF:

T-CNF: $Y_i = 1.287 + 0.0006\, X_i$ $p < 0.0001$

C-CNF: $Y_i = 1.100 + 0.0008\, X_i$, $p < 0.0001$

E-CNF: $Y_i = 1.361 + 0.0012\, X_i$ $p < 0.0001$

A Bonferroni pairwise comparison of means test showed that the highest dose (1000 µg/ml) of T-CNF ($p = 0.0012$) and C-CNF ($p < 0.0001$), as well as the two highest doses of E-CNF (500 and 1000 µg/ml, $p = 0.0014$ and $p < 0.0001$, respectively) were significantly different than the corresponding untreated controls. No differences between doses were found for U-CNF.

For the fine fraction, the ANCOVA revealed a non-significant F*D interaction. However, both F and D were significant ($p<0.0001$), meaning that there was a significant linear dose-response regression for all types of surface functionalization, but the regression coefficient (slope, 0.0009) was the same in all the cases:

U-CNF: $Y_i = 0.971 + 0.0009\, X_i$ $p < 0.0001$

T-CNF: $Y_i = 1.278 + 0.0009\, X_i$ $p < 0.0001$

C-CNF: $Y_i = 1.139 + 0.0009\, X_i$ $p < 0.0001$

E-CNF: $Y_i = 1.330 + 0.0009\, X_i$ $p < 0.0001$

A Bonferroni pairwise comparison of means test showed that, for all types of functionalization, the three highest dose (250, 500 and 1000 µg/ml) were significantly different than the corresponding untreated controls ($p = 0.0103$, $p < 0.0001$ and $p < 0.0001$, respectively).

Moreover, with respect to differences between the types of functionalization, a Bonferroni pairwise comparison means test showed that U-CNF, which had the lowest average value (1.11 RFU), significantly differed from the other types of functionalization ($p = 0.0103$). On the other hand, E-CNF, which showed the highest average value (1.49 RFU), also significantly differed from C-CNF ($p = 0.0084$).

ROS induction (6 h)

As shown in Table I, there was a significant F*D interaction for all the three size fractions. For the coarse and fine fractions, the significant F*D interaction was justified by a significant linear dose-response for all types of functionalization, although the slopes of the regression lines (regression coefficients) differed in each case. On the other hand, the significant F*D interaction for the medium fraction was justified by a significant linear dose-response for all types of functionalization except U-CNF.

The corresponding regression lines were, for the coarse fraction:

U-CNF: $Y_i = 1.396 + 0.0014\ X_i$ $p < 0.0001$

T-CNF: $Y_i = 1.612 + 0.0009\ X_i$ $p = 0.0002$

C-CNF: $Y_i = 2.063 + 0.0039\ X_i$ $p < 0.0001$

E-CNF: $Y_i = 2.016 + 0.0020\ X_i$ $p < 0.0001$

A Bonferroni pairwise comparison of means test showed that, for all types of functionalization, the highest dose (1000 µg/ml) was significantly different than the corresponding untreated controls ($p = 0.0028$ for T-CNF, and $p < 0.0001$ for the other types of

functionalization). For U-CNF, C-CNF and E-CNF, the dose of 500 µg/ml was also significantly different than the zero dose ($p = 0.0126$, $p < 0.0001$ and $p = 0.0006$, respectively). In addition, the dose of 250 µg/ml significantly differed from the untreated controls for C-CNF ($p = 0.0017$).

For the medium fraction:

T-CNF: $Y_i = 1.722 + 0.0011\, X_i$ $p<0.0001$

C-CNF: $Y_i = 1.344 + 0.0019\, X_i$ $p<0.0001$

E-CNF: $Y_i = 1.806 + 0.0025\, X_i$ $p<0.0001$

A Bonferroni pairwise comparison of means test showed that the two highest doses (500 and 1000 µg/ml) were significantly different than the corresponding untreated controls for C-CNF ($p = 0.0023$ and $p < 0.0001$, respectively) and E-CNF ($p < 0.0001$ in both cases). Besides, the highest dose significantly differed from the zero dose for T-CNF ($p = 0.0003$). No differences between doses were found for U-CNF.

And for the fine fraction:

U-CNF: $Y_i = 1.292 + 0.0013\, X_i$ $p < 0.0001$

T-CNF: $Y_i = 1.660 + 0.0018\, X_i$ $p = 0.0002$

C-CNF: $Y_i = 1.491 + 0.0018\, X_i$ $p < 0.0001$

E-CNF: $Y_i = 1.757 + 0.0025\, X_i$ $p < 0.0001$

A Bonferroni pairwise comparison of means test showed that for all types of functionalization, the two highest doses, 500 ($p = 0.0172$ for U-CNF, $p = 0.0024$ for T-CNF, $p = 0.0018$ for C-CNF, and $p < 0.0001$ for E-CNF) and 1000 µg/ml ($p < 0.0001$ for all types of functionalization) were significantly different than the corresponding untreated controls.

ROS induction (24 h)

The F*D interaction was also statistically significant for all the size fractions (Table I). The F*D interaction was justified by a significant linear dose-response for all types of functionalization in the case of the coarse and fine fractions, whereas significant regression did not exist for U-CNF of the medium fraction.

The corresponding regression lines are, for the coarse fraction:

U-CNF: $Y_i = 2.940 + 0.0036\ X_i$ $p < 0.0001$

T-CNF: $Y_i = 3.018 + 0.0026\ X_i$ $p < 0.0001$

C-CNF: $Y_i = 4.460 + 0.0088\ X_i$ $p < 0.0001$

E-CNF: $Y_i = 3.936 + 0.0062\ X_i$ $p < 0.0001$

A Bonferroni pairwise comparison of means test showed that the three highest doses (250, 500 and 1000 µg/ml) were significantly different than the corresponding untreated controls for C-CNF ($p = 0.0018$, $p < 0.0001$ and $p < 0.0001$, respectively) and E-CNF ($p = 0.0134$, $p < 0.0001$ and $p < 0.0001$). Besides, the two highest doses (500 and 1000 µg/ml) of U-CNF also significantly differed from the zero dose ($p = 0.0051$ and $p < 0.0001$, respectively). For T-CNF, the highest dose (1000 µg/ml) significantly differed from the zero dose ($p < 0.0001$).

For the medium fraction:

T-CNF: $Y_i = 3.117 + 0.0032\ X_i$ $p < 0.0001$

C-CNF: $Y_i = 2.394 + 0.0053\ X_i$ $p < 0.0001$

E-CNF: $Y_i = 3.466 + 0.0076\ X_i$ $p < 0.0001$

A Bonferroni pairwise comparison of means test showed that the two highest doses (500 and 1000 µg/ml) were significantly different than the corresponding untreated controls for T-CNF ($p = 0.0123$ and $p < 0.0001$, respectively), C-CNF ($p = 0.0002$ and $p < 0.0001$, respectively) and E-CNF ($p < 0.0001$ in both cases). Besides, the dose of 250 µg/ml

significantly differed from the zero dose for E-CNF ($p = 0.0060$). No differences between doses were found for U-CNF.

And for the fine fraction:

U-CNF: $Y_i = 2.595 + 0.0037\ X_i$ $p < 0.0001$

T-CNF: $Y_i = 3.193 + 0.0043\ X_i$ $p < 0.0001$

C-CNF: $Y_i = 2.909 + 0.0034\ X_i$ $p < 0.0001$

E-CNF: $Y_i = 3.552 + 0.0073\ X_i$ $p < 0.0001$

A Bonferroni pairwise comparison of means test showed that for all types of functionalization, the two highest doses, 500 ($p = 0.0046$ for U-CNF, $p = 0.0012$ for T-CNF, $p = 0.0077$ for C-CNF, and $p < 0.0001$ for E-CNF) and 1000 µg/ml ($p < 0.0001$ for all types of functionalization) were significantly different than the corresponding untreated controls. Besides, the dose of 250 µg/ml significantly differed from the zero dose for E-CNF ($p = 0.0045$).

DNA damage

As shown in Table I, the significant three-way F*D*S interaction was justified by the existence of a significant F*D interaction for the fine fraction, whereas non-significant F*D interaction exist for the coarse and medium fractions, nor a significant Dose covariate. The latter means that none of the functionalization types showed a significant linear dose-response. Hence, a two-way ANOVA, analyzing the effect of F and D, was applied for the latter fractions to find out whether any of the doses significantly differ from the zero dose. In the case of the coarse fraction, only F showed a significant effect ($p = 0.0002$). A Bonferroni pairwise comparison of means test showed that there was no significant difference between the non-anionic U-CNF and E-CNF and between the anionic T-CNF and C-CNF, whereas the anionic CNFs had a significantly ($p<0.01$) higher mean percentage of DNA in tail than the non-anionic CNFs.

On the other hand, neither F nor D showed a significant effect for the medium fraction.

Finally, the significant F*D interaction detected for the fine fraction was justified by a significant dose-response regression for E-CNF:

$$Y_i = 3.124 + 0.0059\ X_i \qquad p < 0.0001$$

A Bonferroni pairwise comparison of means test was applied to assess whether any of the E-CNF doses significantly differ from the corresponding zero dose. The highest two doses, 333 and 1000 µg/ml, showed a significant effect ($p = 0.0029$ and $p < 0.0001$, respectively).

The other types of functionalization of the fine fraction did not show a significant linear dose-response. Hence, a two-way ANOVA was applied to them, showing no significant effect of D or F*D whereas F showed a significant effect ($p = 0.0024$). A Bonferroni pairwise comparison means test showed that T-CNF, which had the highest average value (4.4 % of DNA in tail), significantly differed from the other types of functionalization ($p = 0.0024$).

Chromosome damage

As shown in Table I, there was a significant F*D interaction for the coarse and fine fractions, but not for the medium fraction. For the coarse fraction, the significant F*D interaction was justified by a significant linear dose-response ($p<0.0001$) for C-CNF:

$$Y_i = 12.456 + 0.0166\ X_i \qquad p < 0.0001$$

Whereas for the fine fraction, a significant linear dose-response ($p<0.0001$) was found for E-CNF:

$$Y_i = 16.398 + 0.0309\ X_i \qquad p < 0.0001$$

A Bonferroni pairwise comparison of means test showed that the highest dose of the coarse fraction of C-CNF (1000 µg/ml, $p < 0.0001$), and the two highest doses of the fine fraction of E-CNF (333 and 1000 µg/ml, $p = 0.0005$ and $p < 0.0001$, respectively) were significantly different than the corresponding untreated controls.

The other types of functionalization of both size fractions did not show a significant linear dose-response. When a two-way ANOVA was applied, no significant effect of F, D or F*D were found.

For the medium fraction, a two-way ANOVA, analyzing the effect of F, D and F*D, was applied. Only F showed a significant effect ($p = 0.0050$). A Bonferroni pairwise comparison of means test showed that T-CNF, which had the lowest average mean (11.5 MNBNC/2000 BNC), differed from U-CNF and E-CNF ($p = 0.0106$), E-CNF showing the highest average mean (16.6 MNBNC/2000 BNC).

B. Comparison of the original pulp with the nano-sized coarse fraction of U-CNF

In order to investigate whether the toxic response of the cellulose nanofibrils differ from that of the original pulp fibres, a comparison between the pulp and the coarse fraction of the U-CNF was done for each of the toxicological endpoints. In this case, the proposed model is composed with a factor (size, S) with two levels (pulp, as non-nanoscale size, and coarse U-CNF, as nanoscale size) and dose (D) as a covariate variable. The model was analyzed with an ANCOVA, and the most remarkable result was the lack of a significant S*D interaction for all the endpoints ($p > 0.05$). Moreover, the covariate Dose was neither significant for the DNA damage and chromosome damage endpoints.

For the formation of ROS, both S and the covariate D showed a significant effect at the three time points analyzed ($p < 0.0001$). The corresponding linear regression lines are shown in Table SII. As it can be seen at that table, the coarse fraction of U-CNF always showed higher values than the pulp.

Table II Dose-response regression lines and corresponding p-values for intracellular ROS induction at three time points by the pulp and the coarse fraction of U-CNF

Time	Pulp		Coarse U-CNF	
**3 h**	$Y_i = 0.739 + 0.0006\ X_i$	$p < 0.0001$	$Y_i = 1.071 + 0.0006\ X_i$	$p < 0.0001$
**6 h**	$Y_i = 1.051 + 0.0011\ X_i$	$p < 0.0001$	$Y_i = 1.463 + 0.0011\ X_i$	$p < 0.0001$
**24 h**	$Y_i = 1.938 + 0.0027\ X_i$	$p < 0.0001$	$Y_i = 3.125 + 0.0027\ X_i$	$p < 0.0001$

A Bonferroni pairwise comparison of means test showed that the two highest doses (500 and 1000 µg/ml) significantly differed from the untreated controls at 3 h ($p = 0.0144$ and $p < 0.0001$, respectively), 6 h ($p = 0.0065$ and $p< 0.0001$, respectively) and 24 h ($p = 0.0033$ and $p < 0.0001$, respectively) exposure.

In the case of the DNA and chromosome damage endpoints, a two-way ANOVA, analyzing the effect of S and D, was applied. No significant differences between the pulp and the coarse fraction of U-CNF, nor differences between any of the doses and the zero dose, were observed in the induction of DNA or chromosome damage.

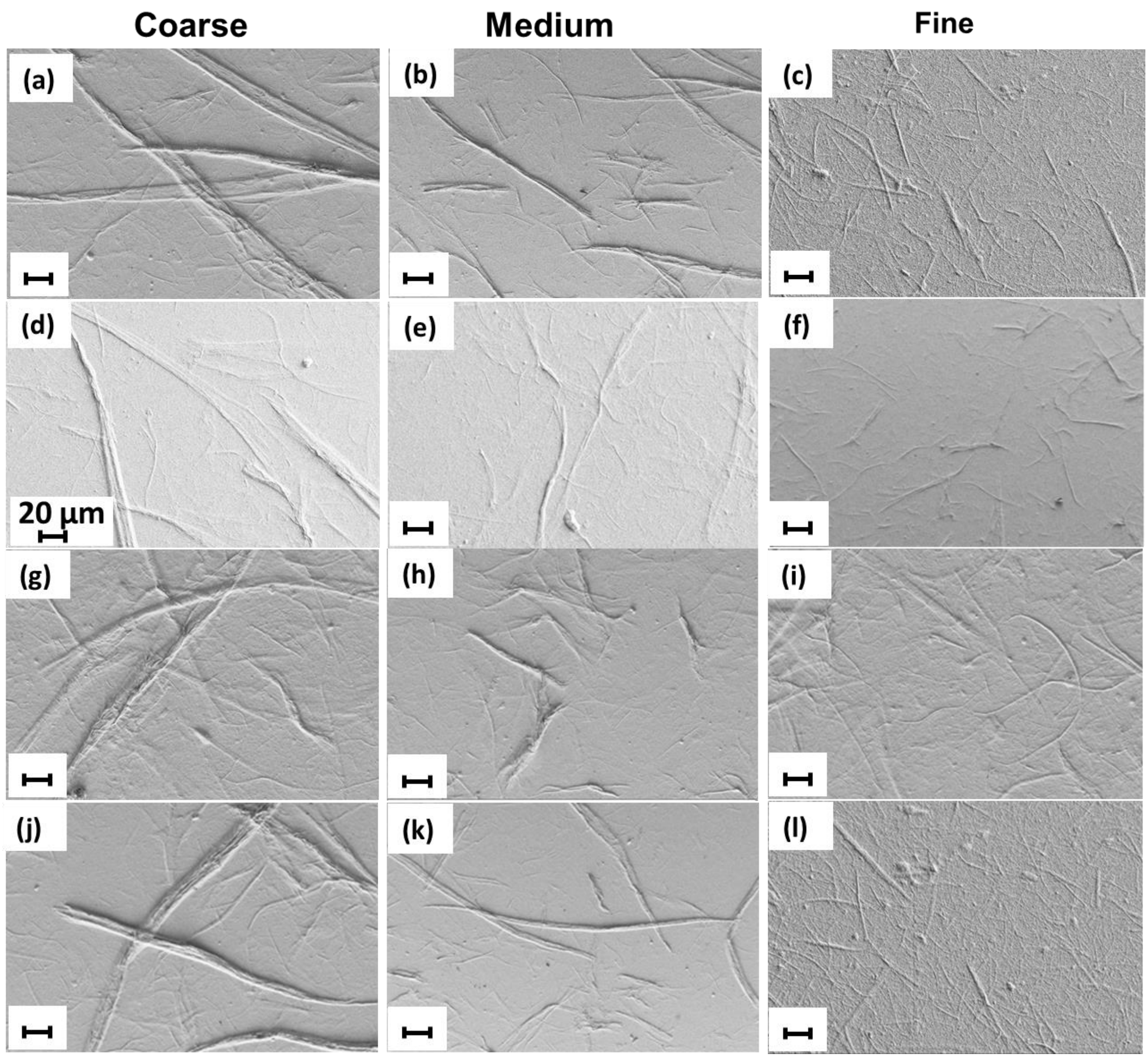

Fig. S1 Field emission scanning electron microscopy images of the three size fractions of U-CNF (a- c), T-CNF (d- f), C-CNF (g-i), and E-CNF (j-l) (N = 100 independent observations).

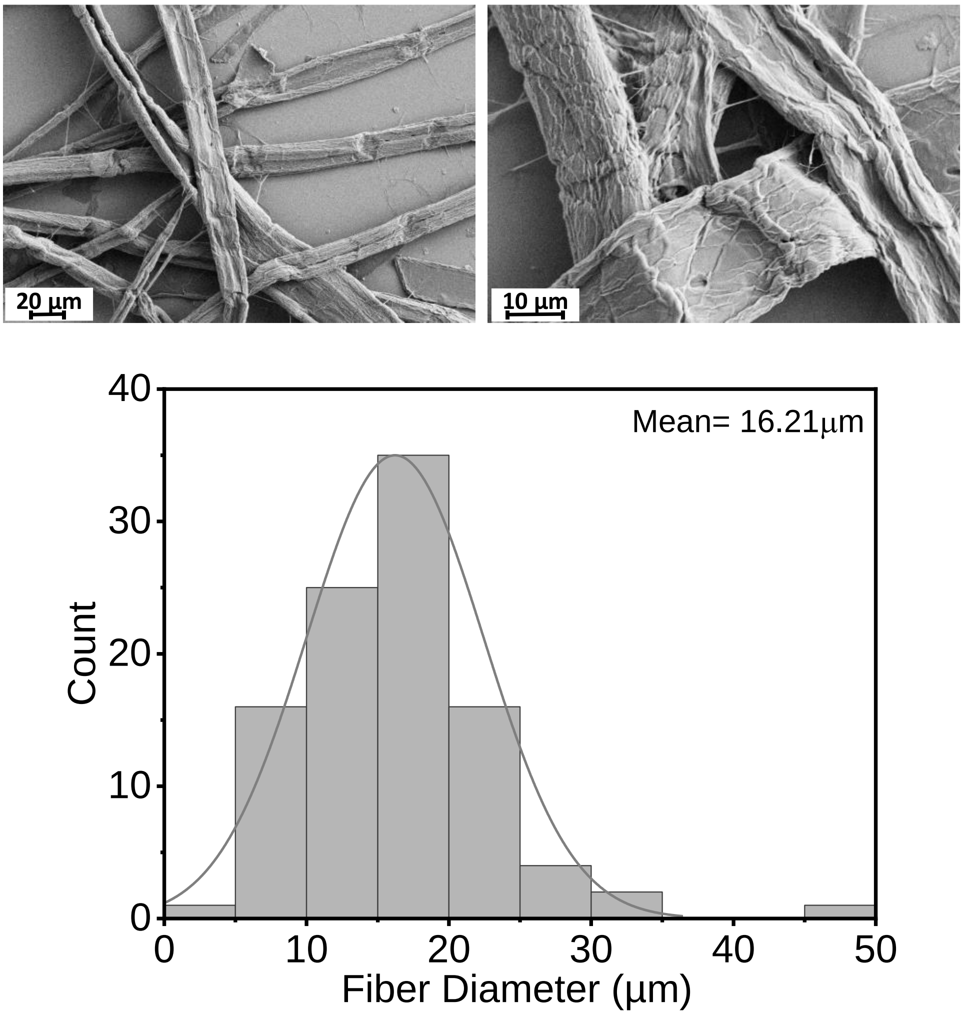

Fig. S2 Scanning electron micrographs and fibre diameter distribution of the birch pulp fibres (N = 100 independent observations).

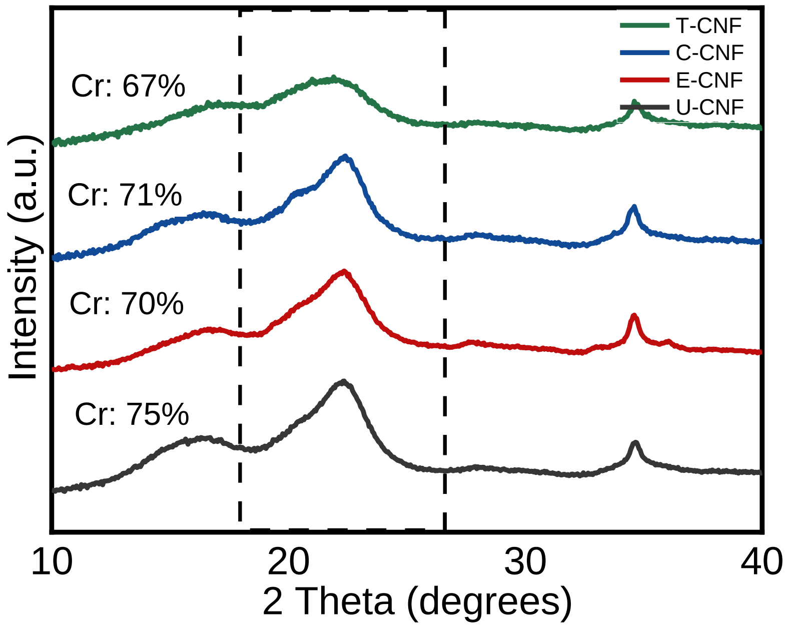

Fig. S3 X-ray power diffraction (XRD) patterns of unfractionated U-CNF, T-CNF, C-CNF and E-CNF samples (N = 2 independent observations).

Table S1 Endotoxin levels measured by the Pierce™ Chromogenic Endotoxin Quant Kit in the pulp source fibres and CNF samples (N = 2 independent replicates).

**Material**	**U-CNF**	**T-CNF**	**C-CNF**	**E-CNF**	**Source fibres**
**Surface modification**	None	TEMPO Oxidation	Carboxy-methylation	EPTMAC quaternization	None
**Size fraction**					
Fine	0.83[a]	> 1.2[a,b]	0.14	0.08	
Medium	0.48	> 1.2[a,b]	0.15	0.07	0.17
Coarse	0.35	> 1.2[a,b]	0.15	0.07	

[a]Levels above the 0.5 EU/ml limit value established by the US Food Drug Agency (FDA) for inhalation studies
[b]1.2 EU/ml was the detection limit allowed by the kit

Table S2 Cellular uptake assessed by the calcofluor staining after a 48-h exposure to 111 µg/ml of the different CNF samples and the source pulp fibres. Data are expressed as mean (± se) percentage of cells showing calcofluor-stained material. No calcofluor-stained material could be found in the untreated cultures (N = 1000 cells per treatment).

**Material**	**Surface modification**	**Size fraction**		
		**Coarse**	**Medium**	**Fine**
U-CNF	None	1.83±0.00	1.93±0.10	0.81±0.00
T-CNF	TEMPO Oxidation	2.70±1.89	2.04±0.62	1.42±0.00
C-CNF	Carboxy-methylation	1.83±0.41	1.22±0.41	2.38±1.78
E-CNF	EPTMAC quaternization	3.63±0.75	2.88±0.21	0.70±0.10
Source fibres	None		1.32±0.31	

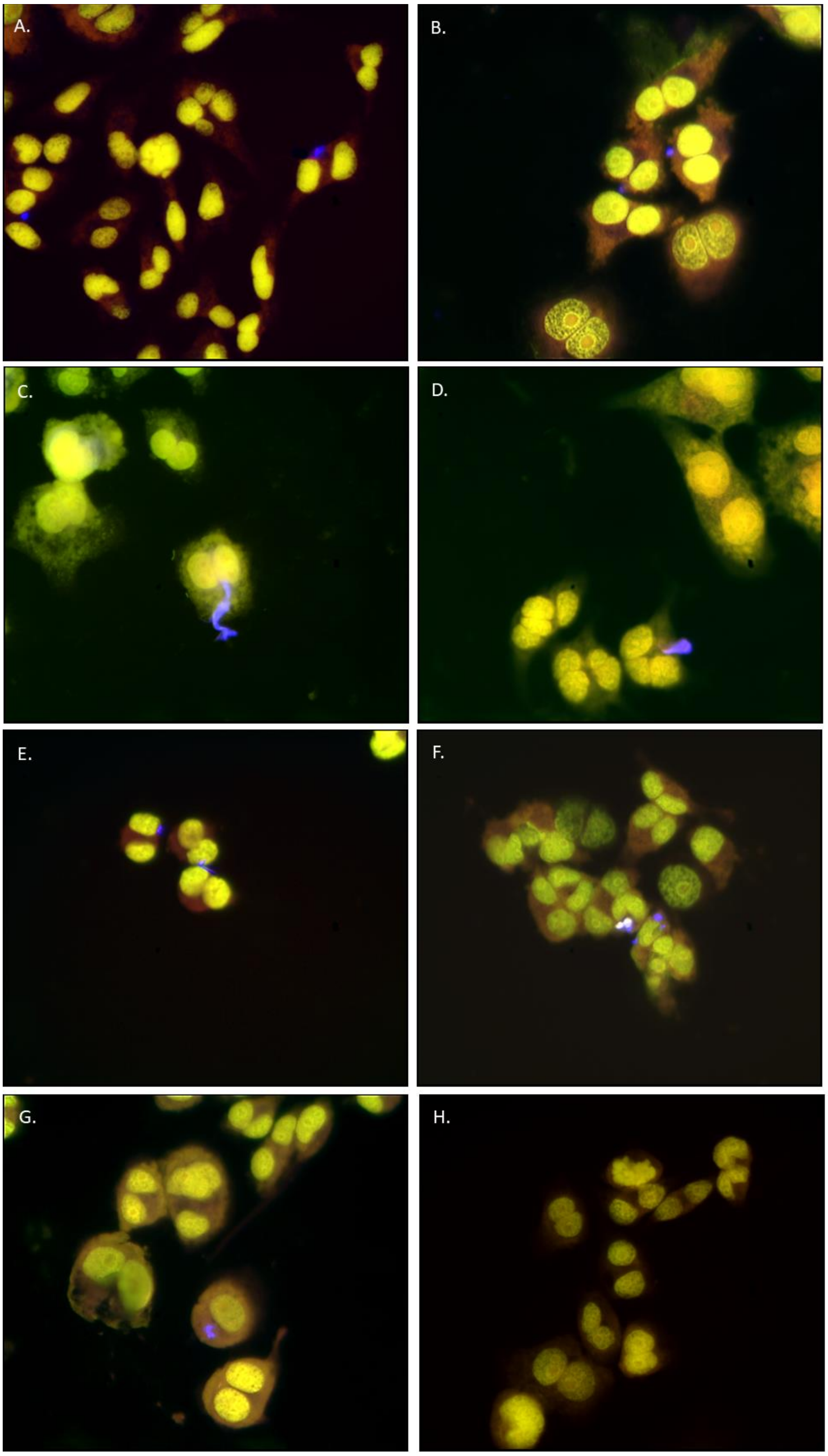

Fig. S4 Calcofluor staining of cellulase pre-treated slides of BEAS-2B cells after a 48-h exposure to 111 µg/ml of the coarse fraction of U-CNF (A), T-CNF (C and G) and E-CNF (E) and of the fine fraction of U-CNF (B), C-CNF (D) and E-CNF (F). Calcofluor-stained CNF appears in blue. Untreated cultures (H) did not show calcofluor staining

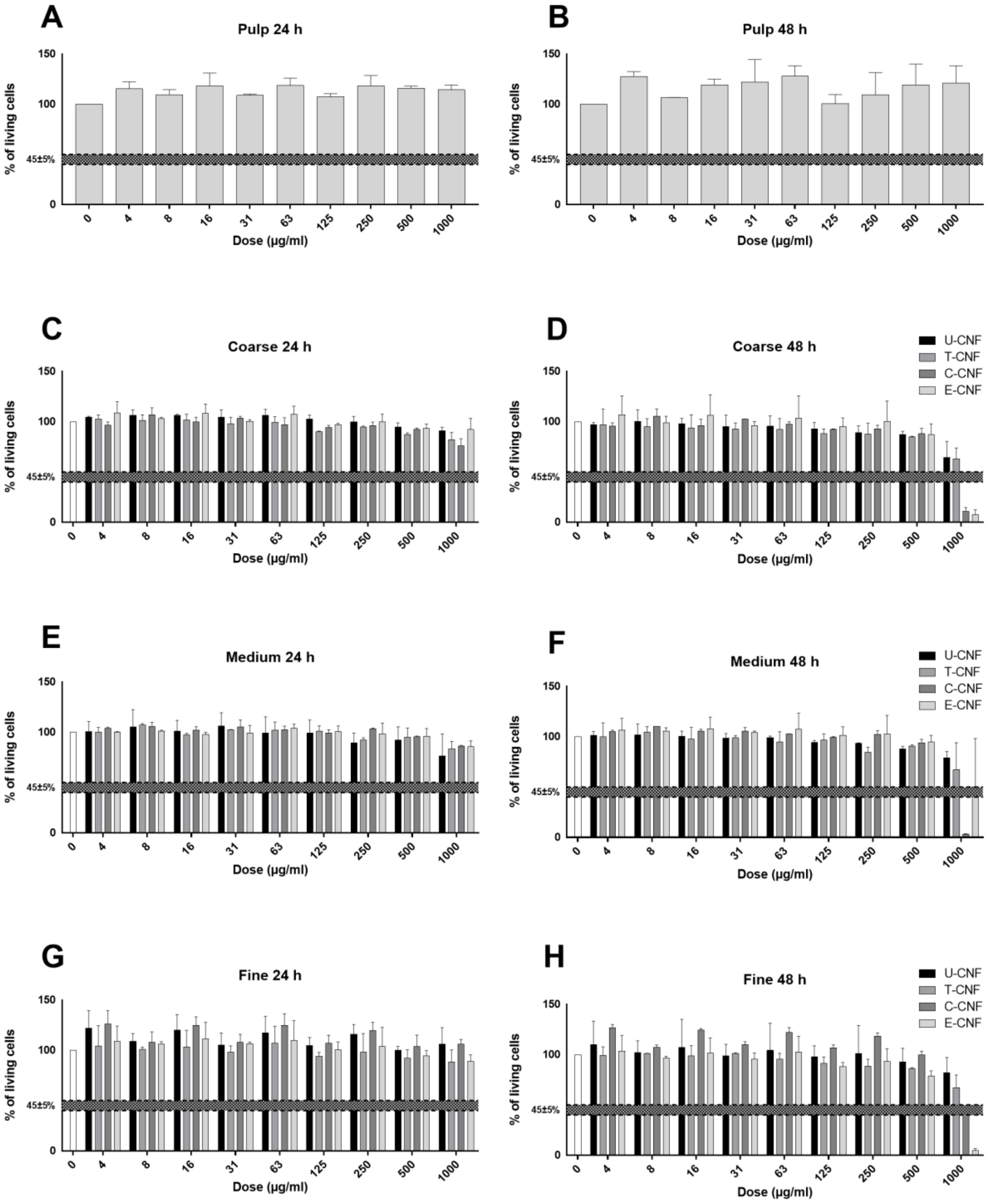

Figure S5 Cell viability (No. living cells as percentage of No. living cells in the control) evaluated by the CellTiter-GloVR Luminescent Cell Viability assay in BEAS-2B cells after 24-h (A, C, E and G) and 48-h (B, D, F and H) exposure to pulp fibres (A and B) and to coarse (C and D), medium (E and F) and fine (G and H) size fractions of cellulose nanofibers (CNF), to define the dose range to be used in the genotoxicity tests. The range of 45±5 % cell viability (corresponding to the 55±5% cytotoxicity) is indicated in each graph. Results are presented as

the mean ± se (N = 2 independent experiments). The positive control, 0.1% Triton X-100, clearly decreased the number of living cells (2.76 ± 0.16 % of living cells in comparison to the negative control) in all the experiments (data not shown)
